# Comparison of Strategies for Typhoid Conjugate Vaccine Introduction in India: A Cost-Effectiveness Modeling Study

**DOI:** 10.1093/infdis/jiab150

**Published:** 2021-11-23

**Authors:** Theresa Ryckman, Arun S Karthikeyan, Dilesh Kumar, Yanjia Cao, Gagandeep Kang, Jeremy D Goldhaber-Fiebert, Jacob John, Nathan C Lo, Jason R Andrews

**Affiliations:** 1 Stanford Health Policy, Center for Health Policy and Center for Primary Care and Outcomes Research, Stanford University School of Medicine and the Freeman Spogli Institute, Stanford, California, USA; 2 Wellcome Trust Research Laboratory, Christian Medical College, Vellore, Tamil Nadu, India; 3 Division of Infectious Diseases and Geographic Medicine, Stanford University School of Medicine, Stanford, California, USA; 4 Department of Community Health, Christian Medical College, Vellore, Tamil Nadu, India; 5 Department of Medicine, University of California, San Francisco, San Francisco, California, USA

**Keywords:** typhoid, enteric fever, vaccines, India, cost-effectiveness, model

## Abstract

**Background:**

Typhoid fever causes substantial global mortality, with almost half occurring in India. New typhoid vaccines are highly effective and recommended by the World Health Organization for high-burden settings. There is a need to determine whether and which typhoid vaccine strategies should be implemented in India.

**Methods:**

We assessed typhoid vaccination using a dynamic compartmental model, parameterized by and calibrated to disease and costing data from a recent multisite surveillance study in India. We modeled routine and 1-time campaign strategies that target different ages and settings. The primary outcome was cost-effectiveness, measured by incremental cost-effectiveness ratios (ICERs) benchmarked against India’s gross national income per capita (US$2130).

**Results:**

Both routine and campaign vaccination strategies were cost-saving compared to the status quo, due to averted costs of illness. The preferred strategy was a nationwide community-based catchup campaign targeting children aged 1–15 years alongside routine vaccination, with an ICER of $929 per disability-adjusted life-year averted. Over the first 10 years of implementation, vaccination could avert 21–39 million cases and save $1.6–$2.2 billion. These findings were broadly consistent across willingness-to-pay thresholds, epidemiologic settings, and model input distributions.

**Conclusions:**

Despite high initial costs, routine and campaign typhoid vaccination in India could substantially reduce mortality and was highly cost-effective.

Typhoid fever is an acute febrile illness that affects millions of people each year worldwide [1, 2]. Typhoid fever is caused by *Salmonella enterica* subspecies *enterica* serovar Typhi (*S.* Typhi) and is spread fecal-orally, primarily through contaminated water and food [1]. Although symptoms can often be mild, severe cases can cause sepsis, intestinal perforation, and other complications that can result in death. More than 100 000 people are thought to die from typhoid-related complications annually [2].

Around half of the global burden of typhoid is concentrated in India [2]. Increasing prevalence of antimicrobial-resistant typhoid in India is a major public health threat. A recent study of *S.* Typhi blood isolates in India found that >80% of isolates were resistant to fluoroquinolones, which have been among the mainstays of therapy in the region [3]. Recent discovery of independently emerging azithromycin-resistant strains has heightened concerns that we will soon run out of effective antibiotic choices [4]. Urbanization also enhances the spread of typhoid, as the concentration of large populations in dense urban informal settlements often outpaces the creation of infrastructure for clean water and sanitation access [5]. In India, urbanization has grown steadily over the past 2 decades and about 1 in 12 Indians lives in slum-like conditions [6]. Although there is substantial geographic variation in typhoid burden within India, urban areas consistently have higher incidence than rural areas in the region [7, 8].

These considerations underscore the need for alternative solutions in the fight against typhoid, such as typhoid vaccines [5, 9]. Historically, adoption of typhoid vaccines in endemic settings has been low due to their modest efficacy, short duration of protection, and lack of approval for use among young children, who are often at greater risk of typhoid in high-burden settings [9, 10]. However, in late 2017, a new typhoid conjugate vaccine (Typbar TCV) was prequalified by the World Health Organization and is now recommended for use in high-burden settings [11]. Typhoid conjugate vaccines are highly immunogenic and have demonstrated high levels of efficacy in a large effectiveness trial, can be safely administered to children as young as 6 months, and are anticipated to cost $1.50 per dose or less [12–16].

The Indian government is considering the introduction of typhoid vaccination to its immunization schedule. However, there are a range of delivery modes and possible targeting by age or geographic setting to consider. Given population heterogeneity in typhoid burden, possible indirect effects of vaccination, the role of asymptomatic carriers, and costs associated with both illness and vaccination, is it not immediately clear whether, and if so which, typhoid vaccination strategies will be good value for money. Previous studies have assessed the cost-effectiveness potential of typhoid conjugate vaccines [17, 18]. However, these studies used preliminary evidence on vaccine efficacy and modeled vaccination across multiple countries, using generalized costing, epidemiologic, and natural history data. Here, we combine data from the first large-scale phase 3 vaccine trial with new evidence on the subnational burden and costs of typhoid fever from a large surveillance study in India to conduct a focused analysis of the cost-effectiveness of typhoid vaccination strategies in India.

## METHODS

### Model Structure

We adapted a dynamic transmission compartmental model governed by a series of differential equations using Julia (version 1.3.1) [19]. Our model allows reductions in infected population sizes caused by vaccination to reduce typhoid transmission, thereby encompassing both direct and indirect benefits of vaccination. We stratified the model by age and urban-rural classification with simulation at the state level, reporting outcomes at monthly intervals. In addition to population inflows and outflows caused by births, aging, and mortality (background and typhoid-specific), we also include migration between rural and urban areas.

### Transmission and Natural History

**Figure 1.**
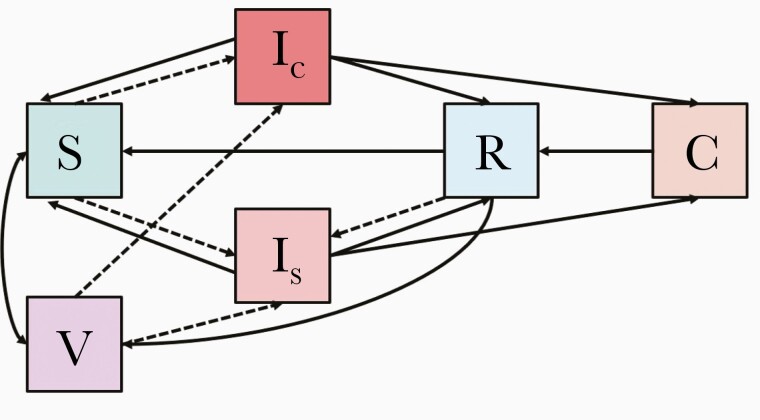
Typhoid infection and natural history. Boxes indicate compartments and arrows indicate transitions between compartments (new infections are further delineated via dashed arrows). Compartment abbreviations are as follows: C, carrier; I_C_, clinically infected; I_S_, subclinically infected; R, recovered; S, susceptible; V, vaccinated. New infections (transitions to the I_C_ or I_S_ compartments) are designated with dashed lines. Details are shown in the Supplementary Technical Appendix.

Our model tracks populations over time based on their age, urban-rural residence, and typhoid status, which we divided into 6 mutually exclusive and collectively exhaustive states (Figure 1). All individuals are born susceptible (S). The probability that those in the susceptible population become clinically (Ic) or subclinically (Is) infected depends on the proportion of the population that is infected and the age-varying transmission rate, β (Supplementary Technical Appendix). The transmission rate is assumed to incorporate both short-cycle (person-to-person) and long-cycle (water-borne) transmission; these 2 routes of transmission are not modeled explicitly for identifiability reasons, as is common in models of typhoid transmission [18]. Because there is increasing evidence that typhoid infection does not always result in durable immunity, our model allows infection to be followed by recovery with medium- to long-term immunity to subsequent typhoid infection (R), recovery without any immunity (transition back to S), or progression to carrier status (C). Carriers continue to be infectious at lower levels of infectivity over a longer duration of time and are unaffected by vaccination. Eventually, carriers recover, upon which they are assumed to be immune (transitioning to R). Susceptible (S) and recovered (R) populations, however, can be vaccinated, upon which they transition to the vaccinated compartment (V), where they can become infected but at substantially reduced susceptibility.

### Vaccination

Vaccination was modeled with high but imperfect immunity that wanes over time, parameterized by data from past and ongoing vaccination trials, including a large phase 3 trial in Nepal (Table 1) [16, 20, 21]. We analyzed 3 main delivery modes: routine vaccination delivered through India’s Expanded Programme on Immunization to 9- to 12-month-olds alongside measles first dose delivery (delivery with measles second dose was explored in sensitivity analysis); routine vaccination plus a 1-time community catch-up campaign targeting 1- to 15-year-olds; and routine vaccination plus a 1-time school-based catch-up campaign targeting school-aged children (aged 5–15 years) with temporary vaccination upon school entry to cover children missed by both the routine and campaign windows (1- to 4-year-olds). We also assessed strategies that target urban populations. Routine vaccine coverage is based on state- and urban/rural-specific data on measles first dose coverage from India’s fourth National Family Health Survey [22] and campaign and school-based vaccination coverage was based on coverage observed in a typhoid vaccine campaign in Mumbai, India (with alternatives explored in sensitivity analysis) [23]. Low levels of vaccination with the lower-efficacy typhoid polysaccharide vaccine were also modeled during the model burn-in period only to reach coverage observed in surveillance [24].

**Table 1.** Model Parameters

Parameter	Mean (95% confidence intervals)	Source
**Transmission and natural history**		
Transmission rate	Varies by state, age group, urban-rural setting	Calibrated (see Supplementary Technical Appendix)
Symptomatic infections (% of total)	10% (6%–21%)	Unpublished estimates from seroprevalence studies
Duration of infection	20 d (12–30 d)	SEFI surveillance data, assume shedding lasts twice as long as symptoms based on data from challenge studies
Case fatality fraction	0.18% (0.07%–0.36%)	SEFI surveillance data; details in Supplementary Technical Appendix
Percentage of infections that mount protective immune response (ie, transition from infected to recovered)	50% (25%–75%)	Estimates from seroprevalence studies, vaccine trial surveillance data, and human challenge studies [25–28]
Duration of immunity against clinical infection	20 y (15–30 y)	Assumed to be the same as duration of immunity from vaccination
Relative infectiousness of subclinical infections	72% (44%–100%)	Control group data from Darton et al [29]
Relative risk of subclinical infection after recent infection	63% (49%–93%)	Calculated from Gibani et al [27]
**Carrier epidemiology**		
Percentage of infections that progress to carrier	0.03%–1.2% (varies by age)	Woodward unpublished report, reported in Gibani et al [27]; Ames et al [30]. See Supplementary Technical Appendix.
Duration of carriage	10 y (5–15 y)	Based on Ames et al [30]; Bhan et al [31], Gunn et al [32]
Relative infectiousness of carriers, compared with acute infections	7.5% (5.5%–9.5%)	Calibrated estimate from Lo et al [17]
**Vaccine characteristics**		
Vaccine efficacy	82% (59%–92%)	Shakya et al [16]
Duration of immunity (TCV)	20 y (15–30 y)	Calculations from seroconversion data from Lanh et al [20] and Bharat Biotech (unpublished data)
Duration of immunity (ViPS)	4 y	Systematic review of vaccine trial data [33]
**Vaccine coverage**		
Routine coverage	47%–98% (varies by state and urban-rural setting)	Measles coverage data from the India National Family Health Survey IV [22]
Campaign coverage	90%	Date et al [23]; alternatives explored in sensitivity analysis
School coverage	90%	
Status quo coverage	6% (4%–8%)	SEFI surveillance data
**Vaccine costs per dose**		
Vaccine	$1.00	Price announcements from the manufacturer [34]
Syringes and safety equipment	$0.031	India Comprehensive Multi-Year Plans [46, 47]
Routine delivery—healthcare costs	$1.47 ($1.31–$1.62)	National Health System Cost Database for India [35]
Routine delivery—out of pocket and time costs	$0	No incremental costs because delivery is alongside measles first dose
Campaign delivery—healthcare costs	$1.53 ($1.40–$4.23)	Date et al [23]
Campaign delivery—out of pocket and time costs	$0.53 ($0.00–$1.49)	Mogasale et al [36]
School-based delivery—healthcare costs	$1.16 ($0.58–$1.74)	Literature review of other school-based vaccination delivery costs [37–40]
School-based delivery—startup healthcare costs	$2.00 ($1.50–$2.50)	Literature review of other school-based vaccination delivery costs [37–40]
School delivery—out of pocket and time costs	$0	No incremental costs because delivery is to children who are already in school
**Cost of illness (for symptomatic infections**)		
Healthcare costs of illness—adult	$20 ($13–$47)	SEFI surveillance data (weighted average of hospitalized and nonhospitalized cases; see Supplementary Technical Appendix and Supplementary Table 2)
Healthcare costs of illness—pediatric	$25 ($13–$37)	
Out of pocket costs of illness	$10 ($7–$17)	
Productivity costs of illness	$56 ($52–$59)	
**Quality of life and disability**		
Severe cases (% of symptomatic infections)	16% (12%–20%)	SEFI surveillance data
Moderate cases (% of symptomatic infections)	84% (80%–88%)	SEFI surveillance data
Ileal perforation (% of severe cases)	2.5% (0.8%–5.1%)	SEFI surveillance data
Duration of symptoms (severe cases)	11.5 d (10.0–13.0 d)	SEFI surveillance data
Duration of symptoms (moderate cases)	9.2 d (8.8–9.5 d)	SEFI surveillance data
Duration of symptoms (severe cases with ileal perforation)	13.4 d (9.6–17.1 d)	SEFI surveillance data
Disability weight (moderate typhoid)	0.051 (0.032–0.074)	Roth et al [41]
Disability weight (severe typhoid)	0.133 (0.088–0.190)	Roth et al [41]
Disability weight (typhoid with ileal perforation)	0.324 (0.220–0.442)	Roth et al [41]
**Demographics**		
Birth rate	Varies by state, urban-rural setting	India SRS bulletin 2019 [42]
All-cause mortality rate and life expectancy	Varies by state, age, urban-rural setting	India SRS life tables 2012–2016 [43]
Urban-rural migration rates	Varies by age	India Human Development Survey 2011–2012 [44]
Population size	Varies by state, age, urban-rural setting	India Census 2011 [45]

Abbreviations: SEFI, Surveillance for Enteric Fever in India; SRS, Sample Registration System; TCV, typhoid conjugate vaccine; ViPS, Vi polysaccharide vaccine.

### Epidemiologic and Demographic Data

Our model incorporated state- and urban-rural–specific typhoid incidence estimates based on geospatial statistical modeling using data from the Surveillance for Enteric Fever in India (SEFI) study, a recent multisite cohort and hybrid surveillance study [8, 24]. Incidence estimates were generated from a geostatistical univariate regression model that was fit to primary SEFI data using the best-fitting model predictor (urban prevalence) chosen from Demographic and Health Survey variables. The statistical model was used to predict typhoid incidence (for all ages combined) at a 5 × 5-km grid level, which was then aggregated to the state urban-rural level with population weighting [8]. We conducted a meta-regression of age-specific incidence data from SEFI, the Surveillance for Enteric Fever in Asia Project (another large ongoing surveillance study in South Asia), and other published active- and hybrid-surveillance studies to calculate a pooled age-incidence relationship that we applied to the modeled incidence estimates to estimate incidence for each state, urban-rural area, and age by 4 groupings (0–4, 5–14, 15–29, and ≥30 years; Supplementary Technical Appendix). SEFI data were also used to estimate duration of illness and the case fatality rate (Supplementary Technical Appendix). Other transmission and natural history parameters came from challenge studies, ongoing serological studies in South Asia, and other published literature [3, 25–32]. We assumed that the average duration of immunity from infection, which is difficult to measure directly, was the same as the average duration of immunity from vaccination, but varied both this parameter and the proportion of infections that mount any immune response widely in sensitivity analysis. Demographic data from India’s Sample Registration System and 2011 census were used to parameterize state- and urban-rural–specific birth rates, age-specific background mortality rates, and the population age distribution [42, 43, 45]. Urban-rural migration estimates came from the 2011–2012 India Human Development Survey [44].

### Model Calibration

We calibrated the transmission rates (β) for 4 age groups (0–4, 5–14, 15–29, and ≥30 years) separately for urban and rural areas within each state to the modeled incidence estimates (targets) for a total of 8 parameters per state. Calibration was conducted by sampling 1000 times from transmission-related parameter distributions and incidence distributions and then, with each sample, conducting directed search optimization using Nelder–Mead with Poisson likelihood-based goodness of fit to identify transmission rates that generated modeled incidence consistent with the sampled incidence targets (Supplementary Technical Appendix; Supplementary Figures 1–2).

### Outcomes and Cost-Effectiveness Analysis

We analyzed costs expressed in 2019 United States dollars (USD) using a societal cost perspective that includes medical and nonmedical vaccination costs, costs of typhoid illness to patients and the healthcare system, and productivity costs from time lost due to illness. We assumed the vaccine costs $1, based on indications from the manufacturer about an India-specific price [34] and estimated injection supply and delivery costs based on published country- and region-specific data and literature on vaccination programs using similar delivery strategies [23, 35–38, 40, 46–49]. Data on the health system and out of pocket costs of symptomatic typhoid illness came from SEFI and were stratified by age (pediatric vs adult) and care delivery setting (hospital inpatient, hospital outpatient, or private clinic/pharmacy; Supplementary Technical Appendix). Productivity loss was monetized by multiplying duration of illness by the average wage, based on International Labor Organization calculations of data from the 2011 Indian National Sample Survey [50]. In additional to cases, typhoid deaths, and costs, we also calculated the disability-adjusted life years (DALYs) associated with each strategy, using disability weights for symptomatic cases from the Institution for Health Metrics and Evaluation [51]. Lifetime DALYs were calculated, based on modeled age and corresponding life expectancy [43].

Our primary outcome of interest was the incremental cost-effectiveness ratio (ICER), defined as the incremental costs of a strategy divided by its averted DALYs, compared to the next most costly nondominated strategy (ie, excluding strategies that yield less health benefit but cost more or have higher ICERs) [52]. The optimal strategy is that with the maximum ICER below a predefined willingness-to-pay (WTP) threshold, after removing dominated and extended dominated options. In consultation with policymakers in India, we used India’s gross national income per capita of $2130 as a WTP threshold, as is standard in many cost-effectiveness analyses conducted in low- and middle-income countries [53]. We discounted both costs and DALYs at a 3% annual discount rate, consistent with recommended guidelines [52]. We considered a 10-year analytic horizon (with lifetime streams of DALYs) but assessed alternative time horizons and WTP thresholds in sensitivity analysis.

### Sensitivity Analysis

We drew 10 000 samples from parameter distributions to conduct a probabilistic sensitivity analysis and calculated main outcomes as averages across samples (with 2.5th and 97.5th quantiles across the 10 000 samples). In addition to the uncertainty over all model parameters that is captured in the probabilistic sensitivity analysis, we used linear regression meta-modeling to describe the influence of individual parameters on results [54]. We also conducted scenario analysis on select parameters, analyzed state-specific results, and included estimates under a healthcare cost perspective.

### Ethics Statement

This project did not meet the definition of human subjects research at Stanford University given use of aggregated estimates of model parameters without identifiable or person-level data. In the primary data collection, all participants provided informed consent with institutional review board approval at Christian Medical College, Vellore.

## RESULTS

### Status Quo Typhoid Burden and Costs

**Figure 2.**
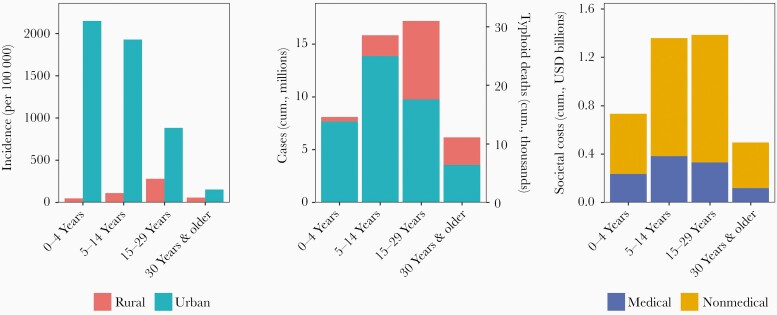
Typhoid outcomes over the next 10 years under the status quo. Left panel displays projected incidence after 10 years of the status quo of no national vaccination strategy, stratified by age group and urban-rural setting. Middle panel displays projected cumulative cases (left axis) and cumulative typhoid deaths (right axis) over the next 10 years, also stratified by age group and urban-rural setting. Right panel shows cumulative costs over the next 10 years, stratified by age group and medical vs nonmedical (out of pocket and productivity) costs. Costs broken down by urban-rural setting are available in Supplementary Figure 3. Abbreviations: cum., cumulative; USD, United States dollars.

Under the status quo of no national vaccination strategy, typhoid incidence is concentrated among younger age groups in urban settings (Figure 2). Annual clinical incidence among children aged <15 years in urban areas is estimated at 2001 cases per 100 000 people, compared with <100 cases per 100 000 people in rural areas and 386 and 138 cases per 100 000 people among adults in urban and rural areas, respectively. However, because two-thirds of India’s population lives in rural areas and three-quarters of the population is aged 15 and older, the numbers of typhoid cases and deaths estimated to occur among older age groups in rural areas are substantial: 1.1 million cases annually in rural areas (3.2 million in urban areas) and 2.1 million cases annually among adults (2.2 million among children). Over the next 10 years, it is expected that there will be 47 million (39–56 million) typhoid cases and 85 000 (36000–163000; 95% credible intervals) deaths in India. The health consequences of typhoid manifest themselves in a considerable economic burden: Cumulative costs of illness in the next 10 years are expected to reach $4.0 billion ($3.2–$4.9 billion) (Supplementary Figure 3). Most (73%) of the costs of illness are non-healthcare costs, stemming largely from lost wages due to time spent sick.

### Cost and Impact of Vaccination

**Figure 3.**
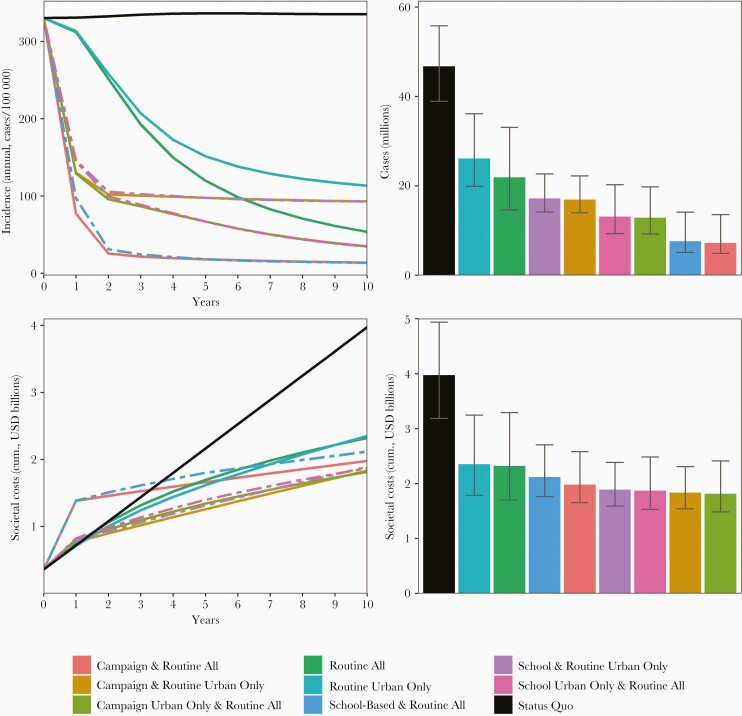
Typhoid incidence and costs over the next 10 years with typhoid vaccination. Left 2 panels show annual incidence (top) and cumulative costs (bottom) by time and strategy over 10 years of implementation. Right 2 panels show annual incidence (top) and cumulative costs (bottom) after 10 years. In the left 2 panels, school-based strategies are shown with dashed lines. Error bars reflect model parameter uncertainty and were calculated based on the 2.5th and 97.5th quantiles of costs and cases across 10 000 simulations. Error bars do not reflect correlation in outcomes across strategies for a given simulation (eg, high cost ranges for 1 strategy typically correlate with high cost ranges for the remaining 8 strategies). Abbreviations: cum., cumulative; USD, United States dollars.

Vaccinating children in urban areas has an outsized impact on incidence, cases, and deaths, due to the concentration of burden among those subgroups. Over the next 10 years, routine vaccination at 9–12 months in urban areas only is expected to yield a 66% reduction in overall incidence, while routine vaccination in both urban and rural areas would reduce incidence by 84% (Figure 3). All strategies that include a campaign (school- or community-based), regardless of urban targeting, have a greater impact on cases and deaths than the routine-only strategies, but routine vaccination in both urban and rural settings has a greater impact on incidence than campaign-based strategies that only vaccinate urban residents. In general, campaigns achieve greater impact by immunizing a wider swathe of the population.

The costs of both typhoid illness and vaccination are expected to be substantial. However, because of the high costs of typhoid illness under the status quo—particularly productivity costs—all vaccination strategies are likely to be cost-saving compared to the status quo by the fourth year of introduction (see Supplementary Figure 4 for healthcare costs only). By year 10, strategies that include nationwide routine and campaign vaccination have the greatest impact on typhoid burden and result in lower net costs than routine vaccination alone but higher net costs than routine and campaign strategies with urban targeting. Results broken down by age and urban/rural setting are available in Supplementary Figure 5.

### Cost-Effectiveness Analysis

All typhoid vaccination strategies are cost-saving compared to the status quo under the societal perspective; several are also cost-saving under the healthcare perspective (Table 2; Supplementary Figure 6). All nondominated strategies (ie, strategies that do not cost more for less health benefit) include routine immunization and a community catch-up campaign, with various degrees of urban targeting. At a WTP threshold of $2130, the preferred strategy under both perspectives is routine vaccination plus a community campaign in both urban and rural areas, which is cost-saving compared to the status quo and has an incremental cost of $929 per DALY averted under the societal perspective and $1812 per DALY averted under the healthcare perspective. Strategies with a school-based catchup campaign achieve slightly less health benefit and cost more than the associated community campaign strategies, but all 6 strategies achieve far greater health benefit than either routine vaccination alone or the status quo.

**Table 2.** Cost-Effectiveness of Typhoid Vaccination Strategies

Strategy	Year 1 Vaccination Costs (USD Millions)	10-Year Cumulative Vaccination Costs (USD Millions)	Discounted Cumulative Costs (Societal, USD Millions)	Discounted Cumulative DALYs Averted (Relative to Status Quo, Millions)	Incremental Cumulative Costs (Societal, USD Millions)	Incremental Cumulative DALYs Averted (Millions)	ICERs (Societal)
Campaign urban only and routine all	271	700	1315	1.488	…	…	Lowest-cost
Campaign and routine urban only	236	383	1317	1.330	Dominated		
School urban only and routine all	312	776	1367	1.474	Dominated		
School and routine urban only	277	460	1369	1.317	Dominated		
Campaign and routine all	794	1222	1530	1.719	214.5	0.231	$929/DALY averted
School-based and routine all	928	1482	1658	1.701	Dominated		
Routine all	56	485	1761	1.061	Dominated		
Routine urban only	21	168	1771	0.899	Dominated		
Status quo	7	39	3125	…	Dominated		

ICERs are shown as incremental cumulative discounted costs per incremental cumulative discounted DALYs averted, compared to the next-highest-cost nondominated strategy. Status quo vaccine costs are nonzero because of the low levels of vaccination under the status quo observed in surveillance data.

Abbreviations: DALY, disability-adjusted life-year; ICER, incremental cost-effectiveness ratio; USD, United States dollars.

### Sensitivity and Uncertainty Analysis

**Figure 4.**
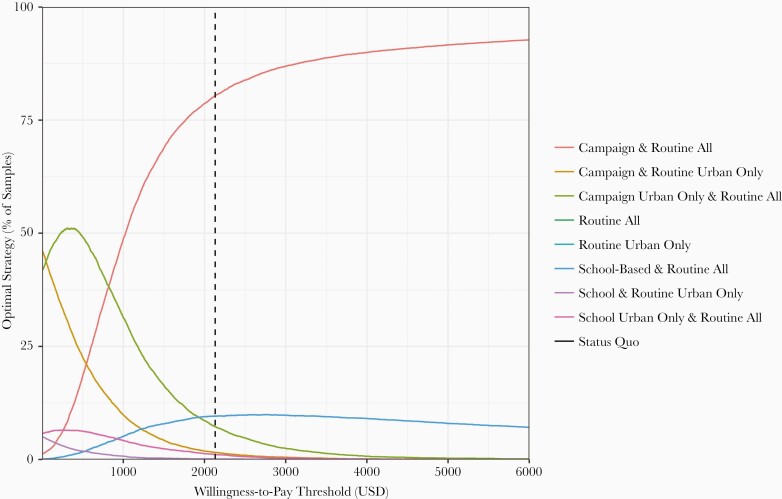
Cost-effectiveness analysis sensitivity over parameter uncertainty and willingness to pay (WTP). Figure shows the proportion of 10 000 probabilistic sensitivity analysis runs for which a given strategy was considered the preferred strategy (nondominated with the highest incremental cost-effectiveness ratio below the WTP threshold) over a range of WTP thresholds. Abbreviation: USD, United States dollars.

These findings are largely robust to parameter uncertainty. At our base case WTP threshold of $2130, the optimal strategy (routine plus community catch-up campaign in urban and rural areas) was the preferred strategy in 80% of probabilistic sensitivity analysis (PSA) samples (Figure 4; Supplementary Figure 7). Routine vaccination with a school-based campaign was preferred in 10% of PSA samples, and in the remainder urban targeting was preferred. Uncertainty decreases at higher WTP thresholds but increases at lower thresholds. However, at all reasonable WTP thresholds, only strategies that include routine vaccination plus a community- or school-based campaign would be considered (the status quo and routine-only strategies are not preferred in any PSA samples).

Under the societal perspective, our findings are consistent across states except for the lowest-incidence settings, where campaigns targeting urban areas are more cost-effective (Supplementary Figure 8). Results are also consistent over longer and shorter time horizons (Supplementary Figure 6) and alternative campaign coverage levels and vaccine prices, which were not varied in the PSA (Supplementary Figures 9 and 10). While vaccine efficacy and duration of protection were varied in the PSA, we also assessed cost-effectiveness under more pessimistic efficacy assumptions and found that although projections of health impact were lower and costs were higher, the preferred strategy did not change (Supplementary Figure 11). Delivering routine vaccination at 15 months (alongside measles second dose) compared to the base case of 9–12 months (alongside measles first dose) would result in slightly higher costs and lower health benefit, although differences are minor (Supplementary Figure 12). These conclusions remain the same under the healthcare perspective, except that campaigns that target urban areas are most cost-effective in several lower-incidence states and would also be preferred at a vaccine price of $1.50 and under an analysis that considers only a 5 year analytic horizon.

**Figure 5.**
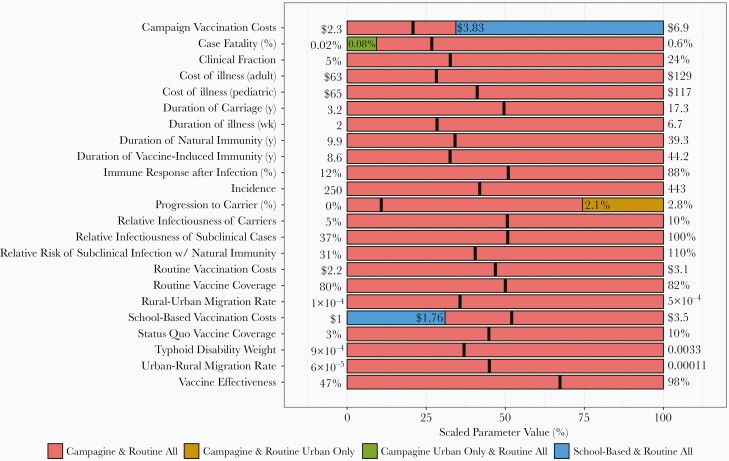
One-way sensitivity analysis. Figure depicts the preferred strategy when all parameters are held at their mean values and a single parameter is adjusted over its full range. Thick black lines indicate the mean values for each parameter. The x-axis indicates the parameter value when it is scaled from 0% to 100%, with 0% representing the minimum, 100% representing the maximum, 50% representing the median, and so on. The x-axis locations where the graph changes color indicate the threshold of that parameter value at which the optimal strategy changes. Parameter minima and maxima are displayed on the plot margins.

For the most part, the preferred strategy did not change when individual parameters were varied over their full range with all other parameters fixed at their mean values (Figure 5). However, the decision was sensitive to 4 parameters: community campaign vaccination costs, school-based vaccination costs, the case fatality rate, and the percent of cases that progress to carriers. When campaign vaccination costs exceed $3.83 per person vaccinated (with school-based vaccination costs held at $2.29 per person vaccinated) or when school-based vaccination costs are less than $1.76 (with campaign costs held at $3.21), school-based campaigns would become preferable to community-based campaigns. School-based vaccination yields slightly lower health benefit at slightly higher cost (because of fewer cases and associated costs of illness averted) compared to community-based campaigns, but at these thresholds the higher relative costs of vaccination associated with a community campaign start to outweigh reductions in the costs of illness.

When case fatality is less than half of the base case value of 0.18%, the health benefits of vaccination are effectively decreased 2-fold, making a more targeted campaign the preferred strategy. Progression to carriage is expected to occur in only 0.3% of cases, but at far higher progression probabilities (≥2.1%), vaccination has less impact and urban targeting would be preferred. Sensitivity analyses under the healthcare cost perspective are shown in Supplementary Figures 13 and 14.

## Discussion

In this model-based analysis, we find that typhoid conjugate vaccines could markedly reduce burden and mortality from typhoid, while averting substantial healthcare and societal costs in India. Targeting young children in urban areas yields sizable incidence reductions, due to the concentration of burden among these subgroups and herd immunity benefits. However, because of the large overall burden and high costs of illness, campaign-based strategies that cover rural areas and older children are likely to be cost-effective across settings that vary in their incidence, costs of and access to quality care, and vaccination coverage and across a range of feasible WTP thresholds. Under our base case analysis, community-based campaigns (with routine vaccination) are the preferred strategy. The health impact of community-based and school-based campaigns is very similar; although community campaigns vaccinate more individuals up front, the inclusion of routine-based vaccination alongside both strategies reduces the incremental impact of this greater initial coverage. The decision between community-based and school-based vaccination and whether to target urban areas may depend on the decision-maker’s WTP threshold and perspective (societal vs healthcare) and could be informed by additional evidence on vaccine delivery costs, case fatality, and carrier progression. Our findings are consistent with previous analyses that have suggested typhoid vaccination campaigns will be cost-effective in highly endemic settings such as India, although our incorporation of the high nonmedical costs of illness makes us the first to conclude that vaccination could also be cost-saving [17, 18].

This analysis drew upon new estimates of typhoid incidence generated from the SEFI project, a multisite prospective study involving both cohort and hybrid surveillance. These data were used to produce subnational typhoid incidence estimates by age, to which our model was then calibrated. In addition, our model incorporated new primary data on the costs of illness, hospitalization, symptoms, and mortality from typhoid. We assessed realistic policies with multiple delivery modes that could feasibly be implemented in India. Finally, our probabilistic sensitivity analysis allowed us to present results that propagated uncertainty in underlying model parameters.

While our analysis incorporated parameter uncertainty to the extent possible, it was difficult to establish evidence-based distributions on some model parameters, particularly those related to typhoid immunity that are difficult to directly measure or may be measurable in human challenge studies only, which are limited in their generalizability to endemic settings. For these parameters, such as the proportion of infections that mount an immune response and duration of immunity from prior infection, we established evidence-based point estimates and allowed these estimates to vary widely in sensitivity analysis. Importantly, in sensitivity analysis we found that the optimal vaccination strategy remained consistent across the full modeled ranges of these parameters. Still, it is possible that the true values of some parameters fall outside of the assigned distributions. We also did not explicitly model both short cycle (person-to-person) and long cycle (water-borne) transmission, instead letting the transmission rate, β, parameterize both. However, there is little reason to expect that vaccination would have a differential impact on these 2 routes of transmission. Additionally, while data on population sizes and migration rates are several years old, results are unlikely to change with gradual shifts in population demographics. Finally, our analysis is subject to common dynamic disease modeling limitations: We assume homogeneous mixing within each urban or rural location within each state, and there were few data against which to perform postcalibration model validation.

Although not included in this analysis, if recent increases in the prevalence of drug-resistant typhoid strains continue, treatment would become less effective, increasing the costs and mortality from typhoid and thus increasing the benefits and cost savings from vaccination [55]. Careful surveillance of typhoid drug resistance in settings where the vaccine is already being introduced, such as Pakistan, can provide evidence that can be incorporated in subsequent analyses. Additionally, no available typhoid vaccines confer protection from paratyphoid fever, which accounts for 10%–20% of enteric fever in the region [2].

Despite these uncertainties, our analysis suggests overwhelmingly that typhoid vaccine introduction will be cost-effective and, in the long-run, cost-saving. However, the initial costs of routine and, especially, campaign vaccination will be substantial. India’s entire immunization budget in 2017–2018 was 6864 crore rupees (approximately $1.1 billion in 2019 USD), of which 79% ($900 million) was funded domestically while 21% came from external donors [47]. Projected healthcare costs of vaccination in the first year of introduction range from $21–$56 million for routine vaccination to $794–$928 million for strategies that include campaigns in both urban and rural areas (Supplementary Figure 15). Campaign-based strategies especially are likely to present substantial budgetary obstacles for the government of India, and funding them without external support could require other important and high-value programs to be sacrificed. Gavi has opened a typhoid conjugate vaccine funding window. India is transitioning away from its current Gavi support in 2021; however, the government of India and Gavi are discussing a next phase (2022–2026) of Gavi support, and there is a possibility for support for new vaccines such as typhoid to be included [56, 57]. However, given the ongoing coronavirus pandemic, a large-scale new vaccine introduction could prove challenging in the near term, both for fiscal and other reasons. If campaign-based strategies are infeasible due to budgetary constraints, routine vaccination would still provide substantial health improvements and yield net cost savings compared to the status quo, while requiring substantially lower increases in the government’s vaccination budget.

Typhoid vaccination could avert 38 000–72 000 deaths and result in net cost savings of $1.6 to $2.2 billion in the first 10 years of introduction. Stakeholders and policymakers in India have expressed interest in typhoid vaccine introduction. Our findings support the broad introduction of typhoid vaccines throughout India to address the high health and economic burden of typhoid fever.

## Supplementary Data

Supplementary materials are available at *The Journal of Infectious Diseases* online. Consisting of data provided by the authors to benefit the reader, the posted materials are not copyedited and are the sole responsibility of the authors, so questions or comments should be addressed to the corresponding author.

jiab150_suppl_Supplementary_Technical_Appendix

## Notes

***Acknowledgments.*** We would like to acknowledge the entire National Surveillance System for Enteric Fever study team and those who participated in the study, without whom this analysis would not have been possible.

***Financial support.*** This work was supported by the Bill & Melinda Gates Foundation (grant number OPP1159351). T. R. is supported by the National Science Foundation’s Graduate Research Fellowship and the Stanford Graduate Fellowship in Science and Engineering. N. C. L. is supported by the University of California, San Francisco Department of Medicine.

***Supplement sponsorship.*** This supplement is sponsored by the Christian Medical College Vellore Association.

***Potential conflicts of interest.*** All authors: No reported conflicts of interest.

Presented in part: 2020 Society for Medical Decision Making Annual Meeting (virtual), October 2020. Abstract 13504; and 2020 American Society for Tropical Medicine and Hygiene Annual Meeting (virtual), November 2020. Abstract 454.
