# Supplementary material for: Comparison of Strategies for Typhoid Conjugate Vaccine Introduction in India: A Cost-Effectiveness Modeling Study
Source: J Infect Dis. 2021 Nov 23;224(Suppl 5):S612–24. doi: 10.1093/infdis/jiab150 (PMC8892534; doi:10.1093/infdis/jiab150)
Supplement: jiab150_suppl_Supplementary_Technical_Appendix [file jiab150_suppl_supplementary_technical_appendix.docx]

**SUPPLEMENTARY APPENDIX**

**Technical Appendix**

In this section of the appendix, we provide additional details on the mathematical model for typhoid transmission, select parameters used in the model, and calibration targets and methods.

*Detailed Model Description*

We developed a dynamic transmission deterministic compartment model that is stratified by age (17 age groups), state (29 states and 6 union territories in India^[[1]](#footnote-1)^), and urban/rural location within state. Our model simulates typhoid transmission, infections, carriage, and immunity in a population over ten years. The model is parameterized by a series of ordinary differential equations that were implemented using the *DifferentialEquations* package in Julia. For each state$i$, age$j$, and urban/rural location $k$ there are 6 equations, one for each typhoid-related compartment, which are described below. Subscripts in the equation indicate which parameters vary by state, age, and urban/rural location. We model monthly time steps and allow interactions between age groups in the same state and urban/rural location as well as interactions between urban/rural areas within a state via migration. Aging and migration are not shown for simplicity.

Equation 1 models the change in the susceptible population ($S$) over time ($t$). Entries into the susceptible compartment include births (rate $u_{B_{i,j,k}}$), recovered/immune populations ($R$) who lose immunity with rate $\omega_{r}$, vaccinated populations ($V$) who lose immunity with rate $\omega_{v}$, and both clinically infected ($I_{c}$) and sub-clinically infected ($I_{s}$) populations that do not mount an immune response following infection (recovery rate $\gamma$ with probability $\left( 1-b \right)$). Transitions out of the susceptible compartment are caused by vaccination, deaths, and new infections. The transmission rate per susceptible person is frequency-dependent and depends on the size of the infectious compartments (including clinically infected $I_{C}$, sub-clinically infected $I_{S}$, and carriers $C$) and the transmission rate $\beta$, which is calibrated and varies by state, broad age group (4 groups), and urban/rural location. Sub-clinical infections and carriers are assumed to be less infectious than clinical infections (with relative risk of transmission of $r_{\mathrm{Is}}$ for sub-clinical infections and $r_{c}$ for carriers). Mass vaccination via catch-up campaigns occurs with rate ${vx}_{i,j,k}$ and depends on which strategy (status quo, routine, community campaign, school-based) is modeled. Routine vaccination is modeled as a probability upon aging out of the 0-8 months compartment, since vaccination occurs with measles first dose, which is given at 9 months. Background mortality out of all compartments is modeled with rate $\mu_{i,j,k}.$

[1: Susceptible] ${{dS}_{i,j,k}}/{dt}=u_{B_{i,j,k}}-\beta_{i,j,k}S_{i,j,k}I_{C_{i,k}}-\beta_{i,j,k}S_{i,j,k}I_{S_{i,k}}r_{\mathrm{Is}}-\beta_{i,j,k}S_{i,j,k}C_{i,k}r_{c}+\omega_{r}R_{i,j,k}+\omega_{v}V_{i,j,k}+\left( 1-b \right)\gamma I_{c_{i,j,k}}+\left( 1-b \right)\gamma I_{s_{i,j,k}}-{vx}_{i,j,k}S_{i,j,k}-\mu_{i,j,k}S_{i,j,k}$

Equations 2 and 3 show how the clinically infected ($I_{C}$) and sub-clinically infected ($I_{S}$) populations change each time step. New infections come from the susceptible compartment, as described above. A proportion of new infections $p$ are clinical, and $\left( 1-p \right)$ are sub-clinical. Vaccinated populations ($V$) can also be infected, but at a much lower transmission rate (based vaccine efficacy $\alpha)$. Infections recover at rate $\gamma.$ In addition to background mortality, clinical infections carry an elevated risk of mortality $\mu_{Ic}$. We also model recovered populations to become sub-clinically infected, at a lower rate parameterized by relative risk $h.$ To keep the model identifiable in calibration, we have collapsed short-cycle (person-to-person) and long-cycle (water-borne) transmission, as has been done in other dynamic models of typhoid transmission [2].

[2: Clinically Infected] ${dI_{C_{i,j,k}}}/{dt}=p\left[ \beta_{i,j,k}S_{i,j,k}I_{C_{i,k}}+\beta_{i,j,k}S_{i,j,k}I_{S_{i,k}}r_{\mathrm{Is}}+\beta_{i,j,k}S_{i,j,k}C_{i,k}r_{C} \right]+(1-\alpha)p\left[ \beta_{i,j,k}V_{i,j,k}I_{C_{i,k}}+\beta_{i,j,k}V_{i,j,k}I_{S_{i,k}}r_{\mathrm{Is}}+\beta_{i,j,k}V_{i,j,k}C_{i,k}r_{C} \right]-\gamma I_{C_{i,j,k}}-\left( \mu_{i,j,k}+\mu_{Ic} \right)I_{C_{i,j,k}}$

[3: Sub-Clinically Infected] ${dI_{S_{i,j,k}}}/{dt}=\left( 1-p \right)\left[ \beta_{i,j,k}S_{i,j,k}I_{C_{i,k}}+\beta_{i,j,k}S_{i,j,k}I_{S_{i,k}}r_{Is}+\beta_{i,j,k}S_{i,j,k}C_{i,k}r_{C} \right]+(1-\alpha)\left( 1-p \right)\left[ \beta_{i,j,k}V_{i,j,k}I_{C_{i,k}}+\beta_{i,j,k}V_{i,j,k}I_{S_{i,k}}r_{Is}+\beta_{i,j,k}V_{i,j,k}C_{i,k}r_{C} \right]$

$$+h\left[ \beta_{i,j,k}R_{i,j,k}I_{C_{i,k}}+\beta_{i,j,k}R_{i,j,k}I_{S_{i,k}}r_{Is}+\beta_{i,j,k}R_{i,j,k}C_{i,k}r_{C} \right]-\gamma I_{S_{i,j,k}}-\mu_{i,j,k}I_{S_{i,j,k}}$$

Upon recovery, individuals with clinical and sub-clinical infections have a probability $\theta_{j}$, which varies by age, of becoming long-term carriers and transition to the $C$ compartment (equation 4), where they continue to be infectious at lower rates of infectivity. Carriers recover and transition to the $R$ compartment at rate $\omega_{c}$.

[4: Carriers] ${{dC}_{i,j,k}}/{dt}=\gamma\theta_{j}(I_{C_{i,j,k}}+I_{S_{i,j,k}})-\omega_{c}C_{i,j,k}-\mu C_{i,j,k}$

Transitions into the recovered/immune compartment (equation 5) occur when infected populations recover at rate $\gamma$, where $\frac{1}{\gamma}$ is the average duration of infectiousness (only a proportion $b$ are assumed to mount an immune response) and when carriers recover at a much slower rate $\omega_{c}$, where $\frac{1}{\omega_{c}}$ is the average duration of carriage. Recovered populations lose immunity and transition back to the susceptible compartment at rate $\omega_{r}$, where $\frac{1}{\omega_{r}}$ is the average duration of natural immunity. Additionally, recovered populations can become sub-clinically infected (described above), vaccinated (we assume that even those with natural immunity get their immunity boosted from vaccination), or die based on the background mortality rate.

[5:Recovered/Immune]${{dR}_{i,j,k}}/{dt}=b\gamma(I_{C_{i,j,k}}+{I_{S}}_{i,j,k})+\omega_{c}C_{i,j,k}-h\left[ \beta_{i,j,k}R_{i,j,k}I_{C_{i,k}}+\beta_{i,j,k}R_{i,j,k}I_{S_{i,k}}r_{Is}+\beta_{i,j,k}R_{i,j,k}C_{i,k}r_{C} \right]-\omega_{r}R_{i,j,k}-{vx}_{i,j,k}R_{i,j,k}- \mu_{i,j,k}R_{i,j,k}$

Equation 6 shows transitions in and out of the vaccinated compartment ($V$). Entries include vaccination from the susceptible and recovered compartments. Because it is difficult to distinguish sub-clinical infections and carriers from susceptible populations, we assume that some sub-clinically infected and carrier populations will also receive the vaccine (and include this in cost estimates), but because they will not gain immunity from vaccination this does not result in any transitions between compartments and is thus not included in the differential equations shown here. We model leaky vaccination, so that vaccinated populations can still be infected but are less susceptible to infection (parameterized by vaccine efficacy $\alpha$). Eventually, vaccinated populations recovered at rate $\omega_{v}$, where $\frac{1}{\omega_{v}}$ is the average duration of immunity from vaccination, or die based on the background mortality rate.

[6:Vaccinated] ${{dV}_{i,j,k}}/{dt}={vx}_{i,j,k}(S_{i,j,k}+R_{i,j,k})-(1-\alpha)\left[ \beta_{i,j,k}V_{i,j,k}I_{C_{i,k}}+\beta_{i,j,k}V_{i,j,k}I_{S_{i,k}}r_{Is}+\beta_{i,j,k}V_{i,j,k}C_{i,k}r_{C} \right]-\omega_{v}V_{i,j,k}-\mu_{i,j,k}V_{i,j,k}$

*Incidence Targets*

Incidence estimates for each state and urban/rural location within state used to calibrate the model come from Cao et al. [1]. Because incidence tends to vary across ages within a given location, we also applied an age distribution to the location-specific incidence estimates. We obtained this age distribution via a meta-regression of published incidence studies, incidence data for each site from the SEFI study, and site-specific incidence data from the Surveillance for Enteric Fever in Asia (SEAP) project. We included in the meta-regression all active surveillance and hybrid surveillance (e.g. passive surveillance with adjustments for treatment seeking and reporting, for example from healthcare utilization surveys or similar) studies that determined typhoid diagnosis based on blood culture and reported incidence for at least two age groups that matched the age groups used to stratify incidence in our model - ages 0-4, ages 5-14, ages 15-29, and ages 30 and above – or at least one age group and all-age incidence. We also included studies for age groups that closely matched these four: for example, we included studies that reported incidence for ages 15-24 and pooled these estimates with estimates from studies that reported incidence for ages 15-29. We adjusted for diagnostic accuracy, assuming a blood culture sensitivity of 0.6.

In total, we identified 13 different studies with data from 36 sites that fit our inclusion criteria [3–15]. We implemented Poisson meta-regression with age-group binary variables as covariates and site-specific random effects using the *xtpoisson* command in *Stata* to statistically combine the age-specific estimates from these studies. We ran separate regressions for low-incidence settings and high-incidence settings. The cutoff between low and high incidence was determined based on examination of the data; sites were classified as high-incidence if either unadjusted all-age annual incidence or annual incidence among young children (ages 0-4 years) exceeded 250 cases per 100,000. We also tested models that only included studies from Asian countries but found that models which included African countries too were not markedly different once we controlled for low vs. high incidence. The regression yielded estimates of age-specific incidence, relative to incidence among children aged 0 to 4 years and standard errors on these regression coefficients were estimated via bootstrap. Results are shown in Supplementary Table 1.

**Supplementary Table 1: Age-Incidence Estimates derived from meta-regression for low incidence (<250 cases per 100,000 person-years among children 0-4 years) and high incidence (≥250 cases per 100,000 person years) settings.**

|  | **Incidence Rate Ratio (relative to ages 0-4)** | |
| --- | --- | --- |
| **Age Group (years)** | **Low-Incidence Settings** | **High-Incidence Settings** |
| 0-4 | reference age group | reference age group |
| 5-14 | 2.15 [1.29 – 3.56] | 0.87 [0.64 – 1.19] |
| 15-29 | 5.76 [3.34 – 9.94] | 0.38 [0.16 – 0.87] |
| ≥30 | 1.02 [0.58 – 1.81] | 0.06 [0.02 – 0.14] |

Note: table shows means with 95% confidence intervals in brackets.

*Case Fatality Details*

The proportion of typhoid cases that result in fatalities and the cost of typhoid illness are both calculated from SEFI data. The calculation for case fatality is shown in equations 1-2.

[1] $CFR_{overall}=prop_{hospital}(CFR_{hospital})+\left( 1-prop_{hospital} \right)(CFR_{non-hospital})$

[2] $CFR_{non-hospital}= \frac{CFR_{hospital}}{{(AFI deaths_{hospital}}/{AFI deaths_{non-hospital}})} x \frac{prop_{hospital}}{(1-prop_{hospital})}$

SEFI data provide direct estimates of the in-hospital case fatality rate ($CFR_{hospital}$) and the proportion of clinical cases that are hospitalized ($prop_{hospital}$, from the active surveillance sites). The proportion of typhoid infections that are not hospitalized but result in death is unobserved, so we estimated it based on the in-hospital case fatality rate, adjusted for the ratio of hospitalized to non-hospitalized (e.g. treated at a pharmacy or private clinic) acute febrile illness deaths ${(AFI deaths_{hospital}}/{AFI deaths_{non-hospital}})$, which comes from the SEFI healthcare utilization survey, and adjusted for the proportion of typhoid cases that are hospitalized, $\frac{prop_{hospital}}{(1-prop_{hospital})}$. Based on these calculations and an in-hospital case fatality rate of 0.84% [95% CI 0.34-1.57%], we estimate a non-hospitalized case fatality rate of 0.06% [95% CI 0.01%-0.22%] and an overall case fatality rate of 0.18% [95% CI 0.07-0.36%].

*Cost of Illness Details*

Medical costs of illness are calculated as the weighted average of inpatient, outpatient (either at a hospital or primary/community health center), and non-hospital (e.g. cases that seek treatment at a pharmacy or private clinic) costs, stratified by age (pediatric vs. adult), with weights equal to the proportion of cases that seek treatment in each setting. Under the societal cost perspective, non-medical costs of illness (such as transportation and lodging costs) and productivity costs (e.g. lost wages due to time spent sick) are also included. Details are provided in Supplementary Table 2.

**Supplementary Table 2: Cost of Illness Estimates**

| **Cost Component** | **Pediatric** | **Adult** | **Notes** |
| --- | --- | --- | --- |
| Inpatient Medical Costs | Rs. 8206 [4344-12097] | Rs. 5971 [3950-16313] | Weighted average of district hospital, community health center, and tertiary care facility costs from SEFI data, with 95% CI estimated as the minimum and maximum across these 3 facility types |
| Outpatient Medical Costs | Rs. 899 [660-1459] | Rs. 921 [682-1481] | Mean estimate comes from SEFI data on district hospital costs. 95% CI established based on costs in other types of facilities (primary health center, community health center, tertiary care facility) |
| Pharmacy/Private Clinic Medical Costs | Rs. 200.0 [47.1-352.9] | | Estimated from pre-hospitalization pharmacy costs for hospitalized cases from hybrid surveillance (SEFI) |
| Inpatient Out-of-Pocket Non-Medical Costs | Rs. 1931 [1654-2206] | Rs. 1596 [1310-1895] | Calculated from SEFI data (hybrid surveillance) from follow-up interviews with patients on out-of-pocket costs, including lodging, food, transport, and other costs. |
| Outpatient Out-of-Pocket Non-Medical Costs | Rs. 453 [276-867] | Rs. 543 [316-1089] | Estimated from inpatient out-of-pocket non-medical costs, adjusted for the difference in the proportion of total costs that are non-medical for inpatients vs. outpatients from Poulos et al. [16] |
| Pharmacy/Private Clinic Out-of-Pocket Non-Medical Costs | Rs 5.8 [3.8-7.8] | | Estimated from pre-hospitalization pharmacy costs for hospitalized cases from hybrid surveillance (SEFI) |
| Inpatient Cases | 16.1% [12.1-20.4%] | | SEFI data (active surveillance) |
| Outpatient Cases | 33.9% [19.2-48.7%] | | Calculated as 100% minus percent inpatient and percent not hospitalized |
| Pharmacy/Private Clinic Cases | 50% [35-65%] | | Estimated from SEFI data (hybrid surveillance). 95% CI is based on range across SEFI sites. |
| Duration of Illness (inpatient cases) | 11.5 days [10-13] | | SEFI data (inpatients from active and hybrid surveillance sites) |
| Duration of Illness (non-inpatient cases) | 9.2 days [8.8-9.5] | | SEFI data (moderate typhoid/outpatient cases from active surveillance sites) |
| Value of Lost Time | Rs. 409/day | | Inflation-adjusted average national wage calculated from the 2011 National Sample Survey reported by the International Labor Organization [17]. For pediatric cases, this is interpreted as a caregiver’s lost wages. |

Note: table shows means with 95% confidence intervals in brackets

*Progression to Carriage*

Estimates of the percentage of infections that progress to become carriers came from Woodward, reported in *Gibani et al Clin Infect Disease 2019* [18] and *Ames et al Am J Public Health 1943* [19]. We used the overall probability from Woodward and applied the relative risks by age from Ames. Supplementary Table 3 shows the age-stratified probabilities used in the model.

**Supplementary Table 3: Probability of progressing to carrier following infection, by age**

| Age | Probability |
| --- | --- |
| 0-19 years | 0.03% |
| 20-29 years | 0.24% |
| 30-39 years | 0.51% |
| 40-49 years | 1.01% |
| 50-59 years | 1.16% |
| 60 years and older | 0.90% |

*Model Calibration*

The goal of calibration was to identify age-specific transmission rates, β, that yielded age-stratified model-predicted incidence that was a good fit to incidence target data. We calibrated the model separately for each state and urban-rural setting to state/urban-rural/age-specific incidence targets (see Technical Appendix section on Incidence Targets for details). Calibration was conducted by implementing Directed Search optimization with the Nelder-Mead algorithm, using the *Optim* package in Julia. Our goodness of fit function was the sum of the Poisson log likelihoods of observing the incidence targets, $x$, for each of 4 age groups, $a$, given that the true incidence, $\lambda$, is that predicted by the model for a given parameter set (equation 1).

[1] $GOF(\lambda;x)= \sum_{a=1}^{4} log(\frac{\lambda_{a}^{x_{a}}}{x_{a}!}e^{-\lambda_{a}})$

We jointly sampled 1000 parameter sets from the transmission parameter and incidence target distributions (for each age group, state, and setting). We induced a correlation of 50% across all of the incidence targets using a published method [20]. This was done to avoid sampling (1) high incidence for one state and low incidence for another and (2) high incidence for both states with equal likelihood, as the former would result in high and low incidence values cancelling out to produce a national-level incidence estimate that is closer to mean on average, which would underestimate uncertainty. We then ran optimization with each of the 1000 parameter sets for each state and setting, resulting in a total of 70,000 calibration simulations. We combined successfully calibrated betas from each of the 1000 parameter sets and sampled from these with replacement to generate 10,000 parameter sets used in the full probabilistic sensitivity analysis.

To allow the model to replicate the current state- and setting-specific population-age distributions, prior to calibrating the model’s transmission rates, we ran the model without any typhoid transmission or typhoid cases and calibrated starting (at time=0) age-specific population sizes, age-specific deaths rates, and birth rates, and changes in the birth and death rates over time. These parameters were calibrated so that birth rates, death rates, and population size would closely match demographic data from the India Census and Sample Registration System [21–23]. This demographic calibration was also conducted using Directed Search with Nelder-Mead and the *Optim* package. We then used these burn-in rates and initial compartment sizes when calibrating the model.

**Appendix References**

1. Cao Y, Karthikeyan AS, Ramanujam K, et al. Geographic pattern of typhoid fever in India: a model-based estimate of cohort and surveillance data. **forthcoming**; .

2. Bilcke J, Antillón M, Pieters Z, et al. Cost-effectiveness of routine and campaign use of typhoid Vi-conjugate vaccine in Gavi-eligible countries: a modelling study. Lancet Infect Dis. **2019**; 19(7):728–739.

3. John J, Bavdekar A, Rongsen-Chandola T, et al. Estimating the incidence of enteric fever in children in India: a multi-site, active fever surveillance of pediatric cohorts. BMC Public Health. **2018**; 18(1):594.

4. Saha S, Sayeed KMI, Saha S, et al. Hospitalization of Pediatric Enteric Fever Cases, Dhaka, Bangladesh, 2017–2019: Incidence and Risk Factors. Clin Infect Dis. Oxford Academic; **2020**; 71(Supplement_3):S196–S204.

5. Tamrakar D, Vaidya K, Yu AT, et al. Spatial Heterogeneity of Enteric Fever in 2 Diverse Communities in Nepal. Clin Infect Dis. Oxford Academic; **2020**; 71(Supplement_3):S205–S213.

6. Yousafzai MT, Irfan S, Thobani RS, et al. Burden of Culture Confirmed Enteric Fever Cases in Karachi, Pakistan: Surveillance For Enteric Fever in Asia Project (SEAP), 2016–2019. Clin Infect Dis. Oxford Academic; **2020**; 71(Supplement_3):S214–S221.

7. Breiman RF, Cosmas L, Njuguna H, et al. Population-Based Incidence of Typhoid Fever in an Urban Informal Settlement and a Rural Area in Kenya: Implications for Typhoid Vaccine Use in Africa. PLOS ONE. Public Library of Science; **2012**; 7(1):e29119.

8. Thriemer K, Ley B, Ame S, et al. The Burden of Invasive Bacterial Infections in Pemba, Zanzibar. PLOS ONE. Public Library of Science; **2012**; 7(2):e30350.

9. Sinha A, Sazawal S, Kumar R, et al. Typhoid fever in children aged less than 5 years. The Lancet. Elsevier; **1999**; 354(9180):734–737.

10. Brooks WA, Hossain A, Goswami D, et al. Bacteremic Typhoid Fever in Children in an Urban Slum, Bangladesh - Volume 11, Number 2—February 2005 - Emerging Infectious Diseases journal - CDC. [cited 2020 Dec 1]; . Available from: https://wwwnc.cdc.gov/eid/article/11/2/04-0422_article

11. Naheed A, Ram PK, Brooks WA, et al. Burden of typhoid and paratyphoid fever in a densely populated urban community, Dhaka, Bangladesh. Int J Infect Dis IJID Off Publ Int Soc Infect Dis. **2010**; 14 Suppl 3:e93-99.

12. Ochiai RL, Acosta CJ, Danovaro-Holliday MC, et al. A study of typhoid fever in five Asian countries: disease burden and implications for controls. Bull World Health Organ. **2008**; 86(4):260–268.

13. Owais A, Sultana S, Zaman U, Rizvi A, Zaidi AKM. Incidence of Typhoid Bacteremia in Infants and Young Children in Southern Coastal Pakistan. Pediatr Infect Dis J. **2010**; 29(11):1035–1039.

14. Marks F, Kalckreuth V von, Aaby P, et al. Incidence of invasive salmonella disease in sub-Saharan Africa: a multicentre population-based surveillance study. Lancet Glob Health. Elsevier; **2017**; 5(3):e310–e323.

15. Guiraud I, Post A, Diallo SN, et al. Population-based incidence, seasonality and serotype distribution of invasive salmonellosis among children in Nanoro, rural Burkina Faso. PLOS ONE. Public Library of Science; **2017**; 12(7):e0178577.

16. Cost of illness due to typhoid fever in five Asian countries - Poulos - 2011 - Tropical Medicine &amp; International Health - Wiley Online Library [Internet]. [cited 2019 May 16]. Available from: https://onlinelibrary.wiley.com/doi/full/10.1111/j.1365-3156.2010.02711.x

17. India Wage Report: Wage policies for decent work and inclusive growth [Internet]. 2018 Aug. Available from: http://www.ilo.org/newdelhi/whatwedo/publications/WCMS_638305/lang--en/index.htm

18. Gibani MM, Voysey M, Jin C, et al. The Impact of Vaccination and Prior Exposure on Stool Shedding of Salmonella Typhi and Salmonella Paratyphi in 6 Controlled Human Infection Studies. Clin Infect Dis Off Publ Infect Dis Soc Am. **2019**; 68(8):1265–1273.

19. Ames WR, Robins M. Age and Sex as Factors in the Development of the Typhoid Carrier State, and a Method for Estimating Carrier Prevalence. Am J Public Health Nations Health. **1943**; 33(3):221–230.

20. Goldhaber-Fiebert JD, Jalal HJ. Some Health States Are Better than Others: Using Health State Rank Order to Improve Probabilistic Analyses. Med Decis Mak Int J Soc Med Decis Mak. **2016**; 36(8):927–940.

21. Census of India Website : Office of the Registrar General & Census Commissioner, India [Internet]. [cited 2019 Jun 17]. Available from: http://www.censusindia.gov.in/vital_statistics/SRS_Bulletins/Bulletins.html

22. Census of India Website : SRS Based Life Table [Internet]. [cited 2019 Jun 17]. Available from: http://www.censusindia.gov.in/vital_statistics/Appendix_SRS_Based_Life_Table.html

23. Chandramouli C. Census of India: Provisional Population Totals [Internet]. Goverment of India; 2011 [cited 2020 Aug 1]. Available from: https://censusindia.gov.in/2011-prov-results/paper2/data_files/india/paper2_1.pdf

24. Shakya M, Colin-Jones R, Theiss-Nyland K, et al. Phase 3 Efficacy Analysis of a Typhoid Conjugate Vaccine Trial in Nepal. N Engl J Med. **2019**; 381(23):2209–2218.

**Supplementary Figures**

**Supplementary Figure 1A: Calibrated model fit to incidence targets – urban settings**

**
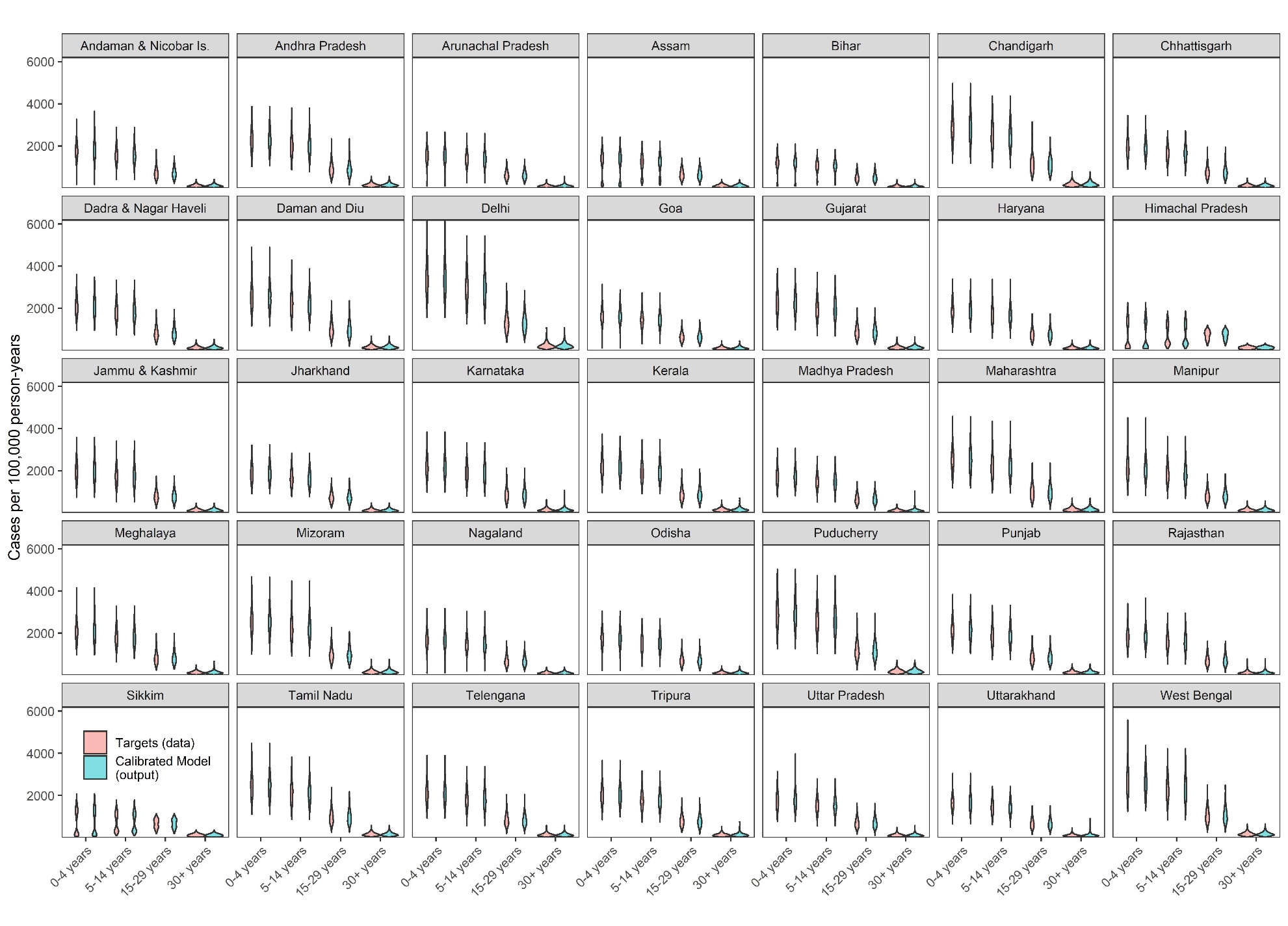
**

**Supplementary Figure 1B: Calibrated model fit to incidence targets – rural settings**


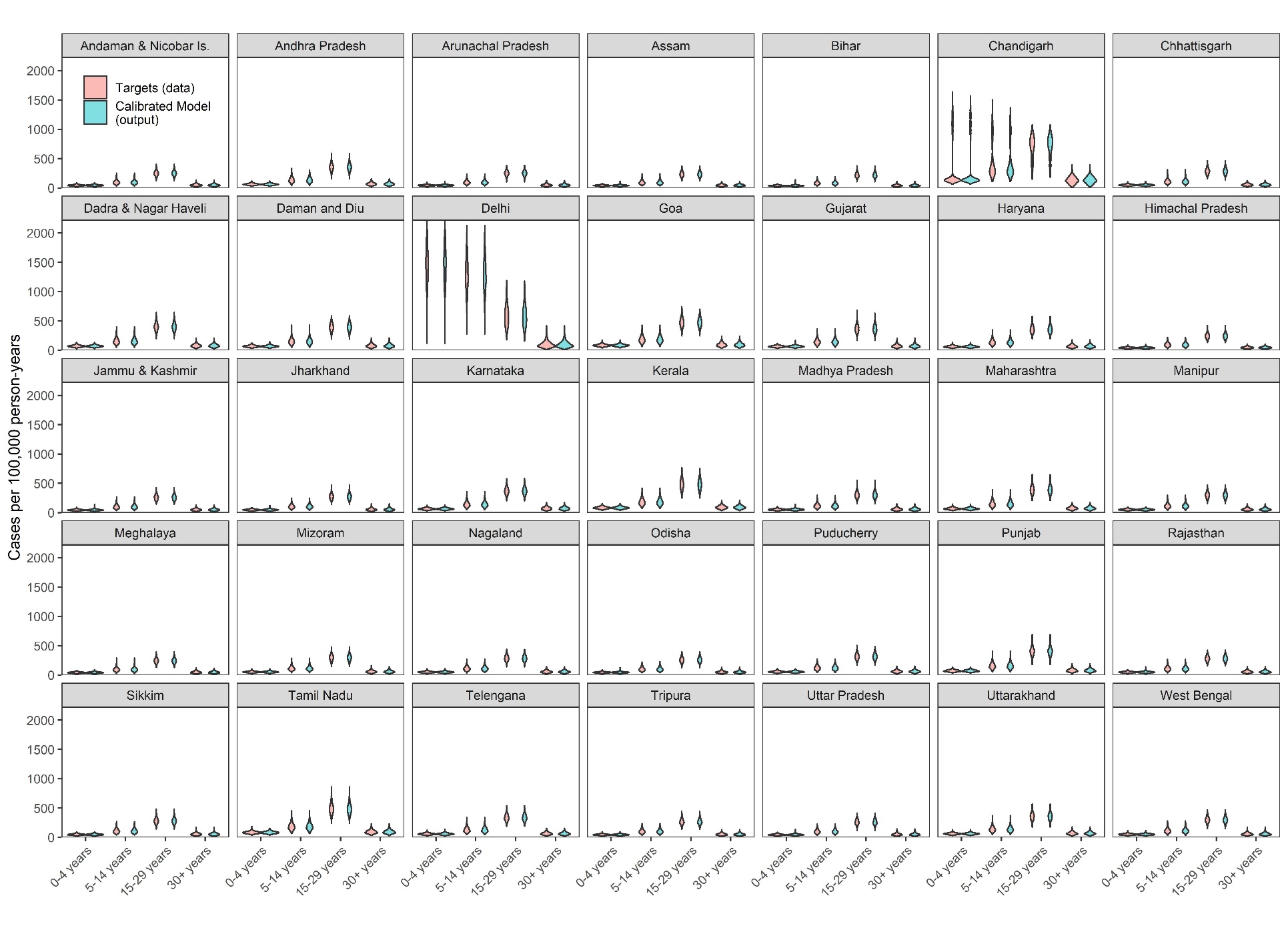


Note: Figure displays the target distributions on all-age incidence from geospatial estimates compared to the estimated incidence from the calibrated model, for each state, in urban areas (panel A) and rural areas (panel B).

**Supplementary Figure 2A: Distributions on calibrated transmission rates – urban settings**


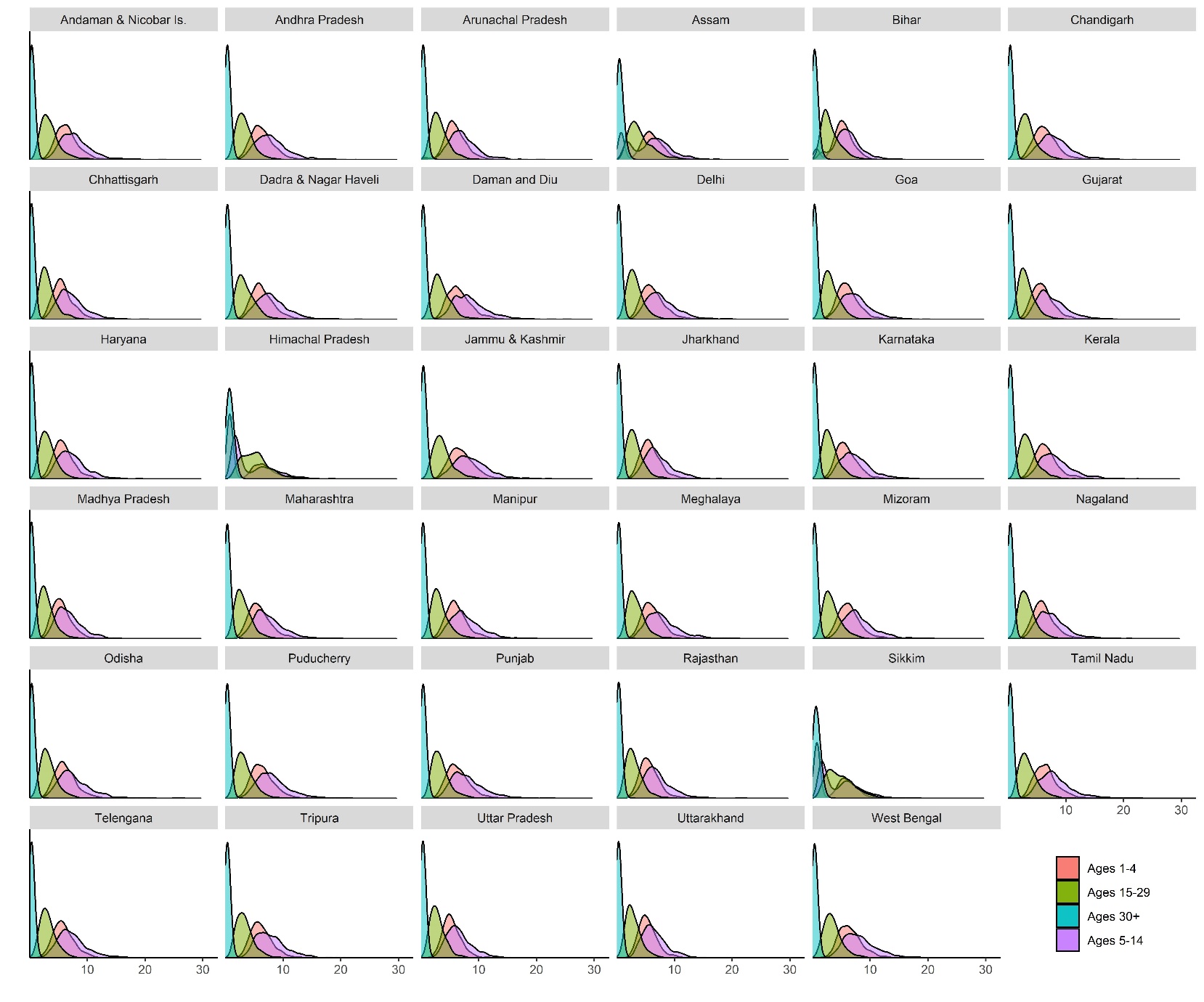


**Supplementary Figure 2B: Distributions on calibrated transmission rates – rural settings**


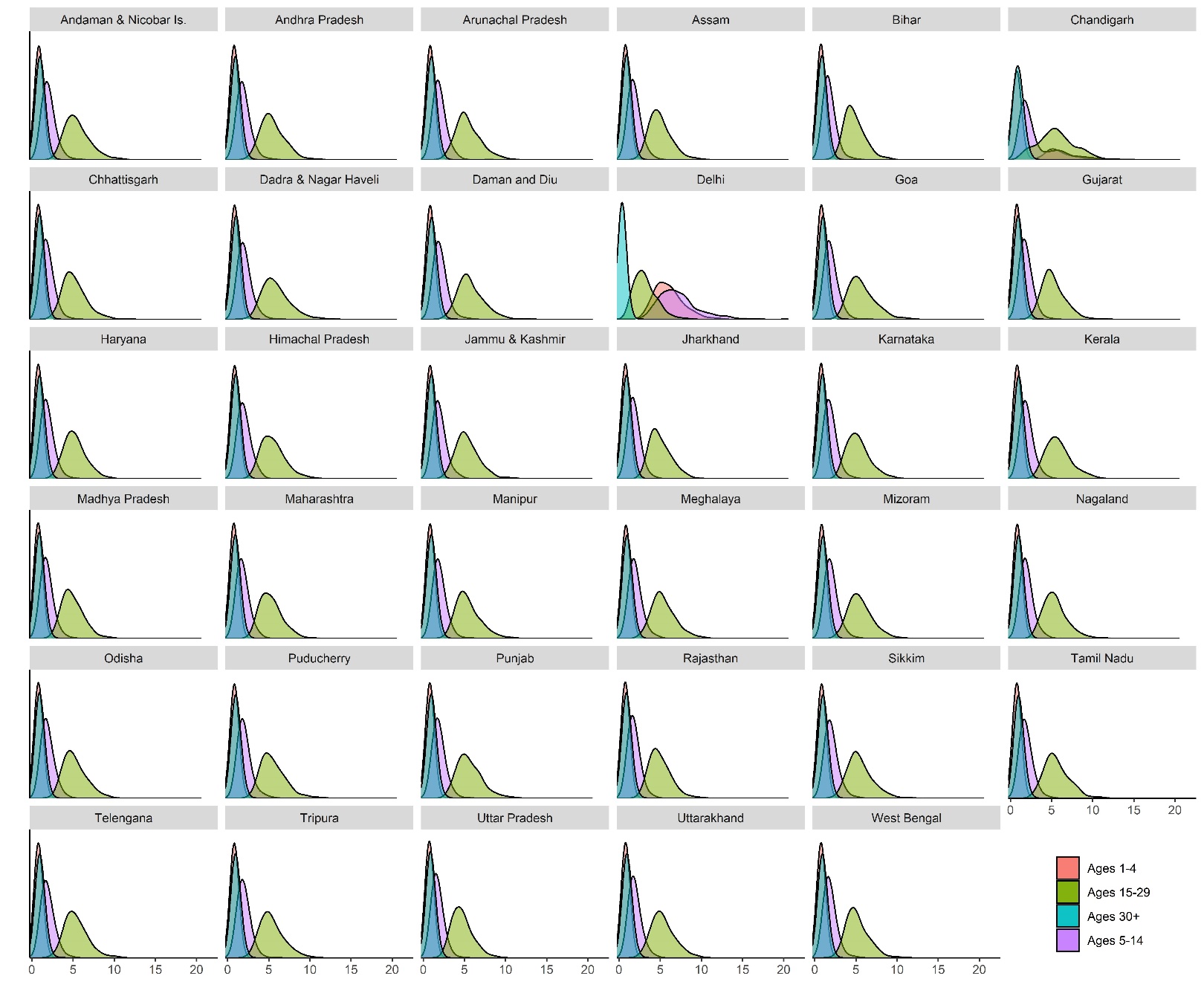


Note: Figure displays the calibrated transmission rate distributions, by age, for each state, in urban areas (panel A) and rural areas (panel B).

**Supplementary Figure 3: Status quo costs by age and setting**

**
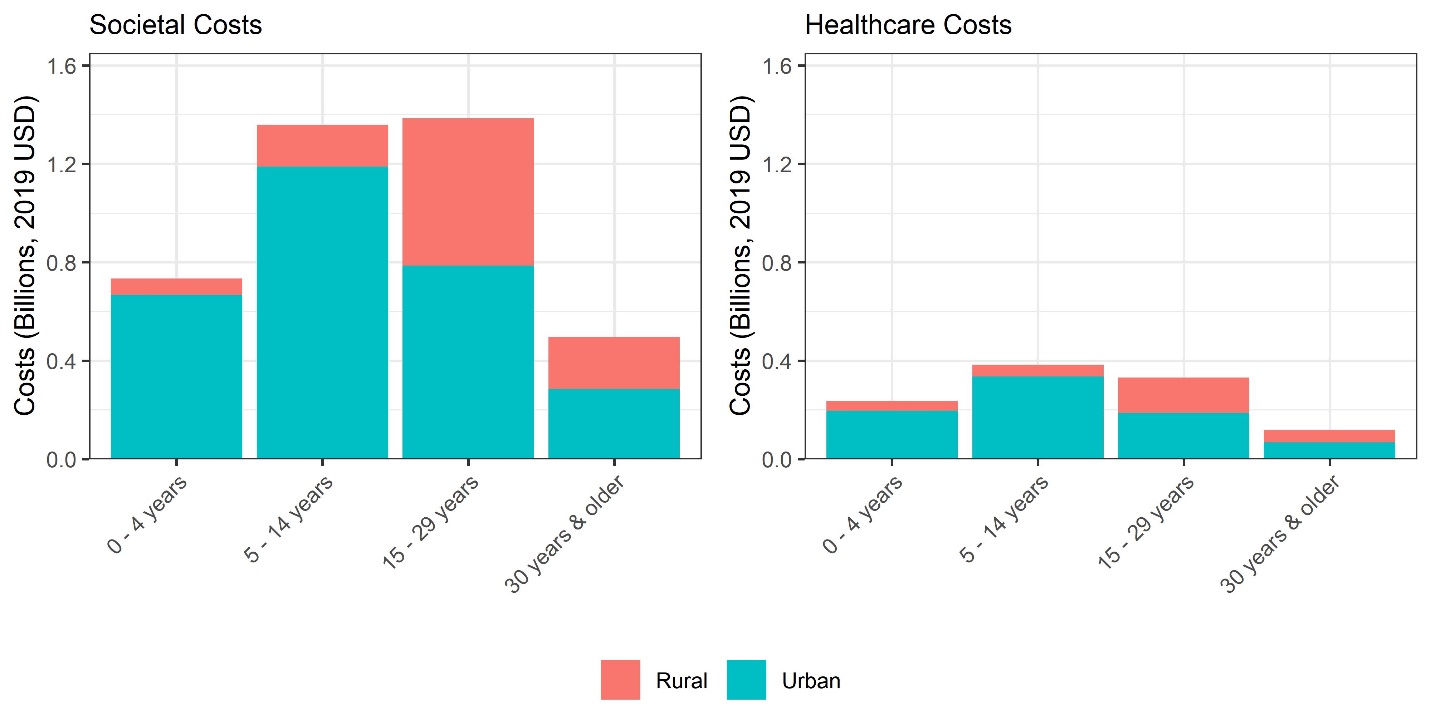
**

Note: Figure displays the cumulative projected 10-year costs under the status quo based on a societal perspective (left panel; includes out-of-pocket non-medical costs and productivity costs in addition to medical costs) and a healthcare perspective (right panel; medical costs only)

**Supplementary Figure 4: Cost projections under societal and healthcare perspectives**

**
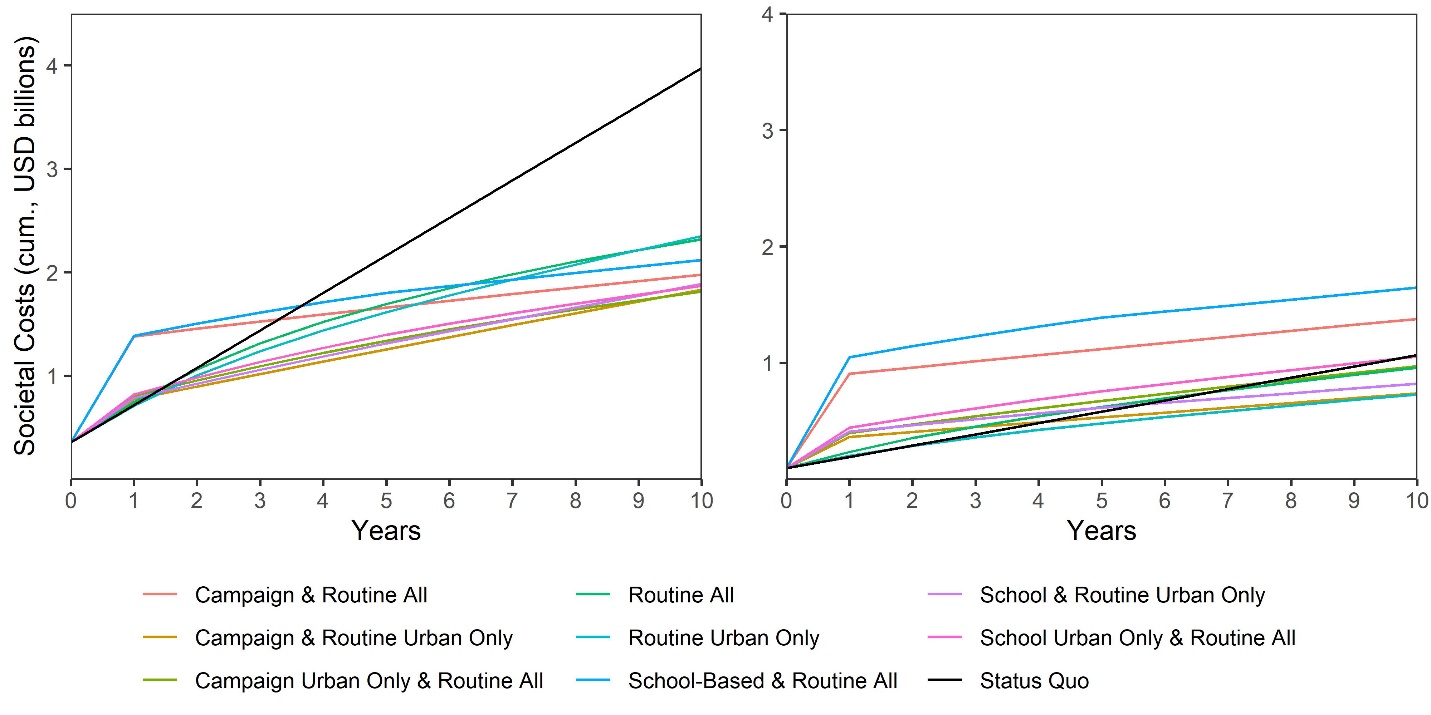
**

Note: Left panel shows cost projections under the societal perspective, right panel shows cost projections under the healthcare perspective.

**Supplementary Figure 5: Case projections by age and setting**

**
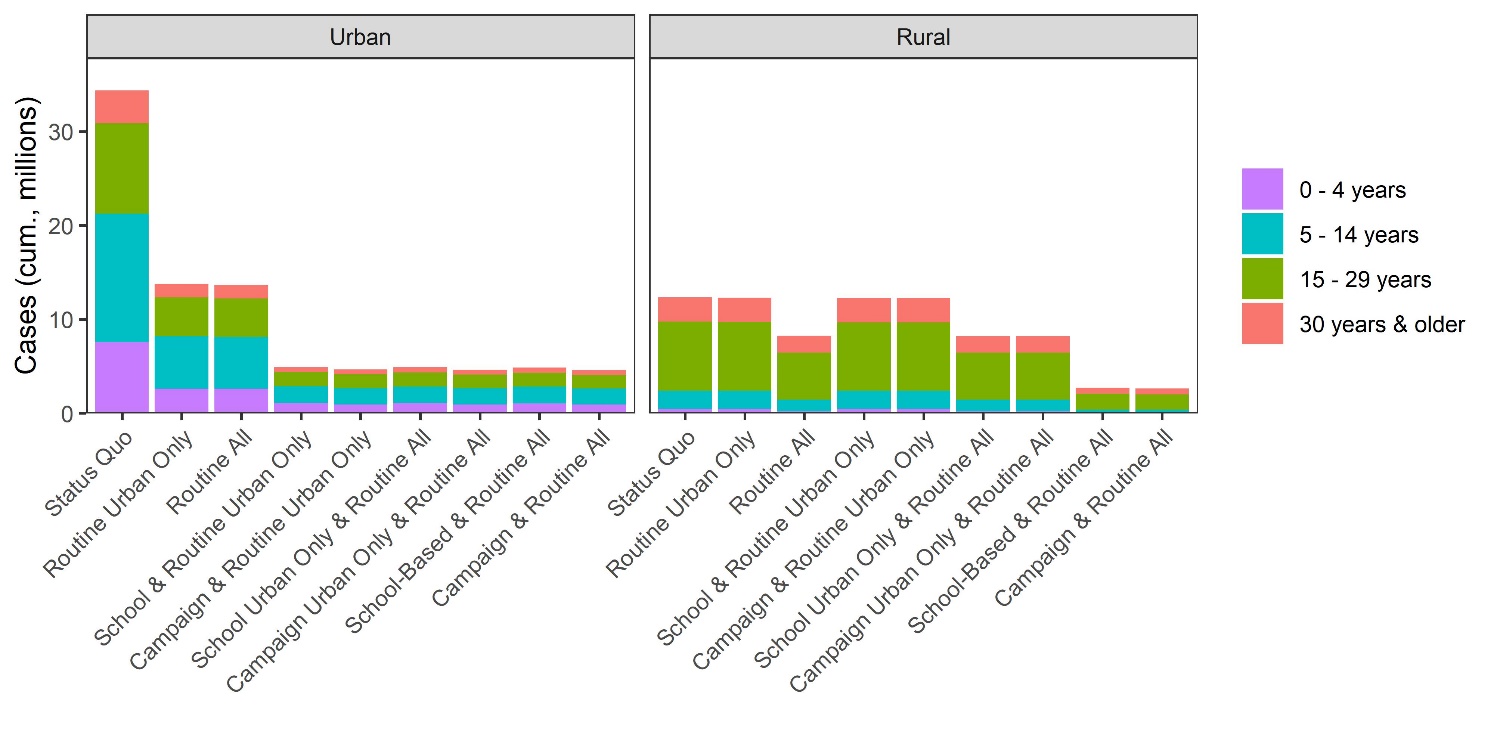
**

Note: Figure displays projected cumulative cases (after 10 years) by strategy, broken down by age and setting.

**Supplementary Figure 6: Cost-effectiveness results under alternate time horizons and perspectives**


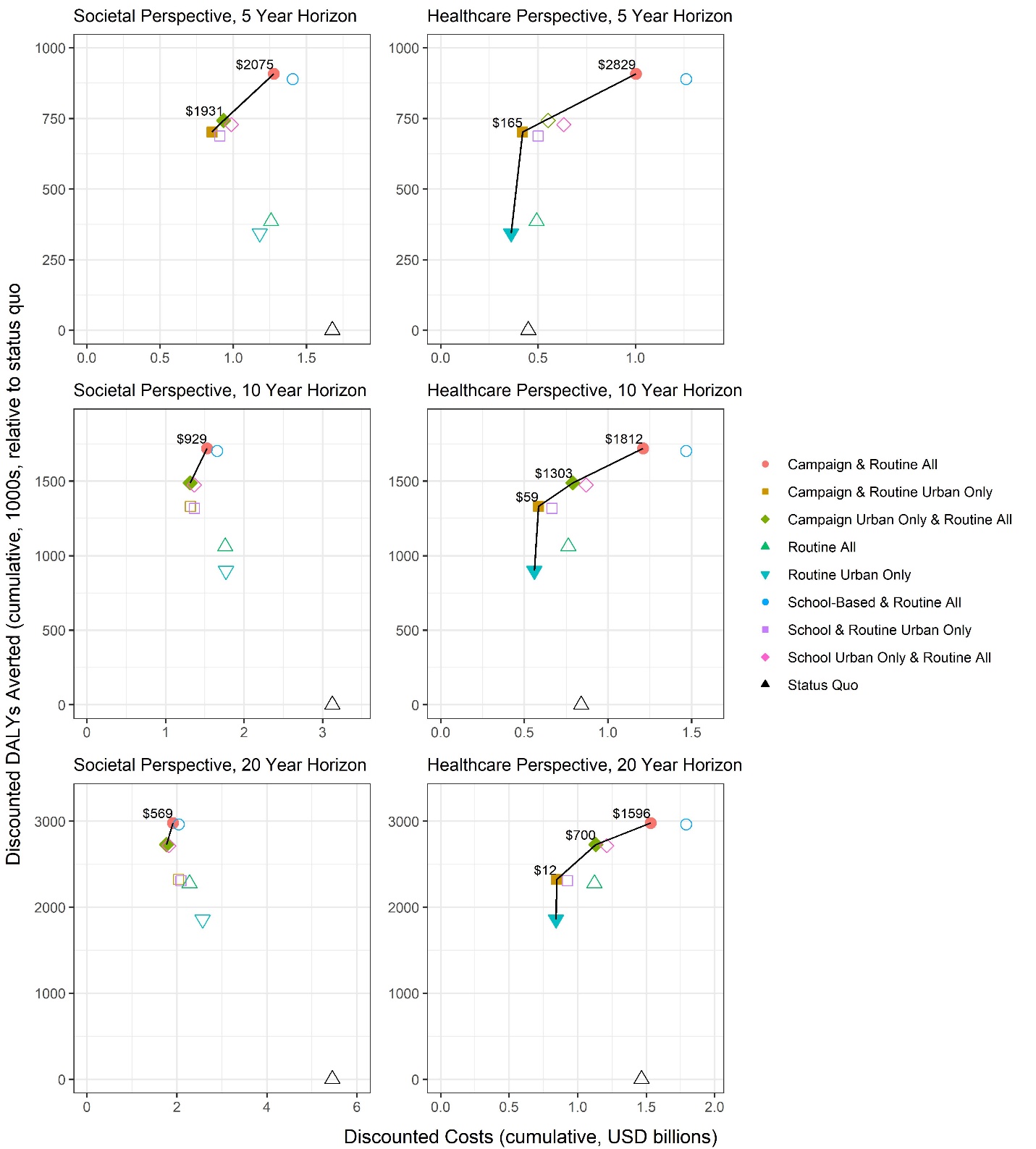


Note: Figure shows cumulative discounted costs (USD billions) on the x-axis and cumulative discounted DALYs averted (thousands) compared to the status quo of no national vaccination strategy. Filled markers indicate those on the cost-effectiveness frontier, while unfilled markers indicate that the strategy is dominated (costs more for less health benefit compared to another strategy). Panels vary by the time horizon (5, 10, and 20 years) and perspective (societal vs. healthcare).

**Supplementary Figure 7: Cost-effectiveness acceptability frontier**

**
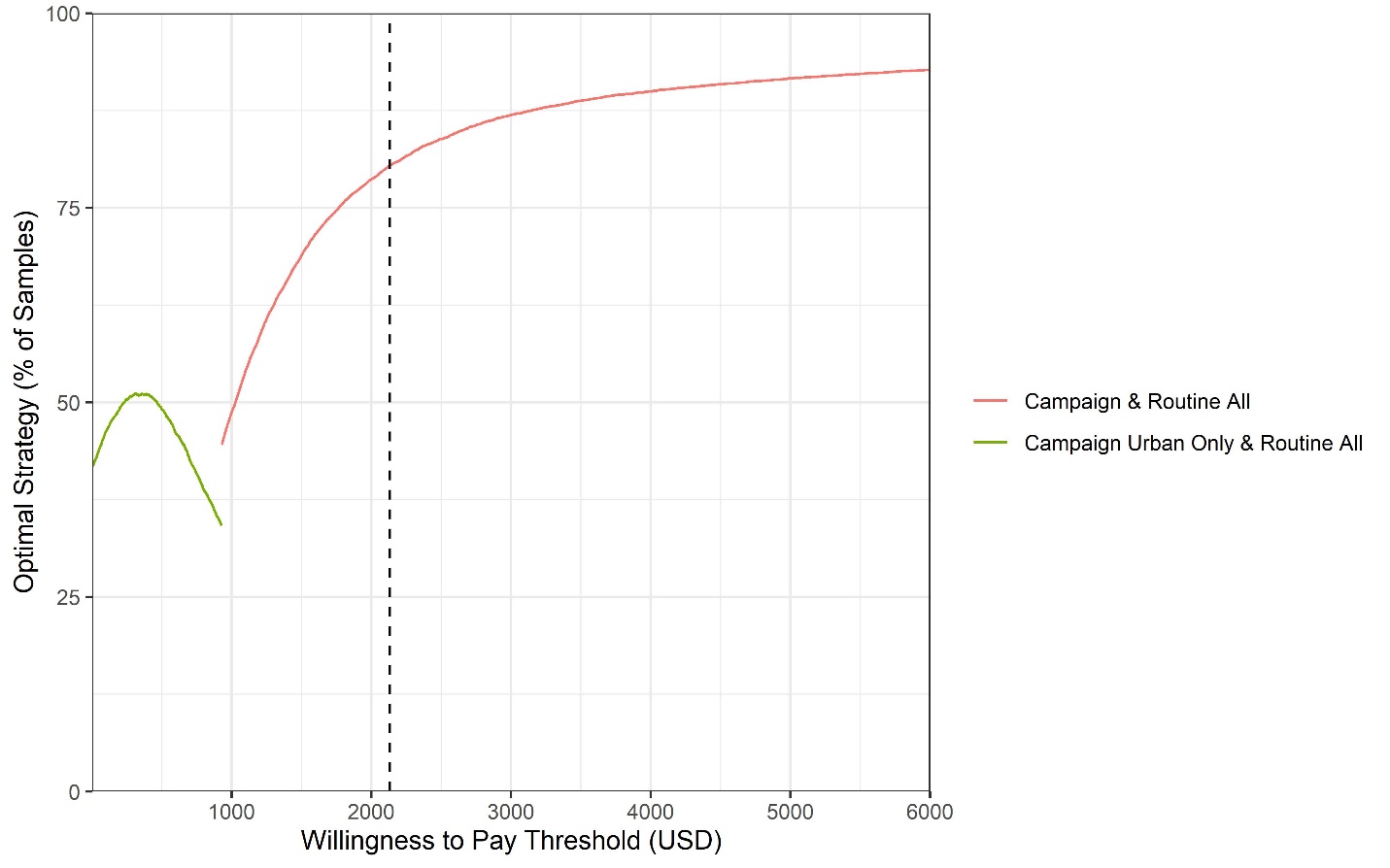
**

Note: The Cost-Effectiveness Acceptability Frontier (CEAF) shows the proportion of 10,000 probabilistic sensitivity analysis runs for which the strategy that was considered preferred on expectation (across all 10,000 runs) was considered the preferred strategy for that run (non-dominated with the highest ICER below the willingness to pay threshold) over a range of willingness to pay thresholds.

**Supplementary Figure 8: State-level cost-effectiveness results**

**
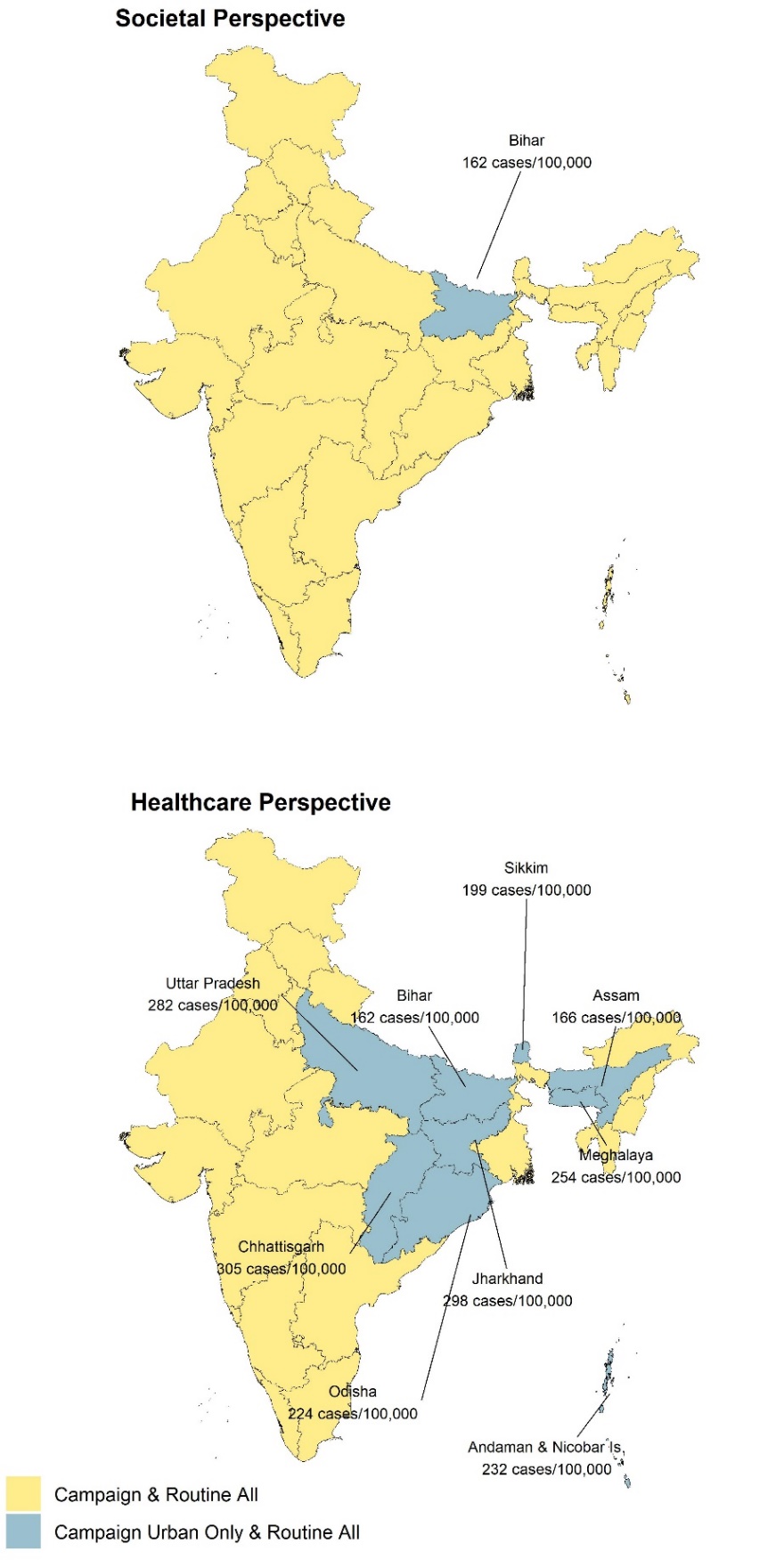

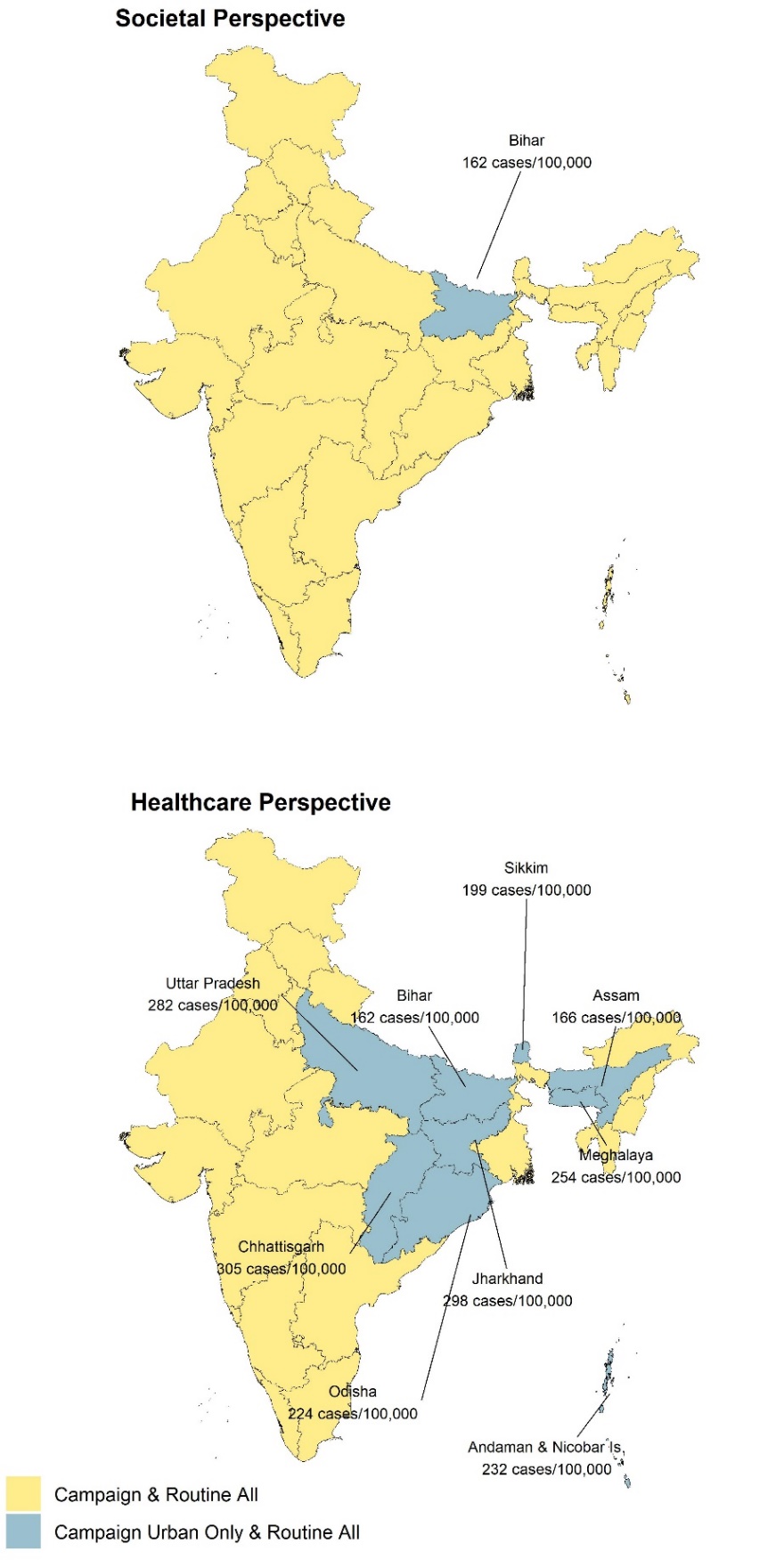
**

Note: Maps are filled based on the preferred strategy in each state, using a WTP threshold of $2310 and 10-year analytic horizon. States with a preferred strategy that differs from the base case national preferred strategy are labeled with mean annual incidence. The designations employed and the presentation of the material on this map do not imply the expression of any opinion whatsoever on the part of the authors concerning the legal status of any country, territory, city or area or of its authorities, or concerning the delimitation of its frontiers or boundaries.

**Supplementary Figure 9: Cost-effectiveness results under alternate vaccine coverage assumptions**

**
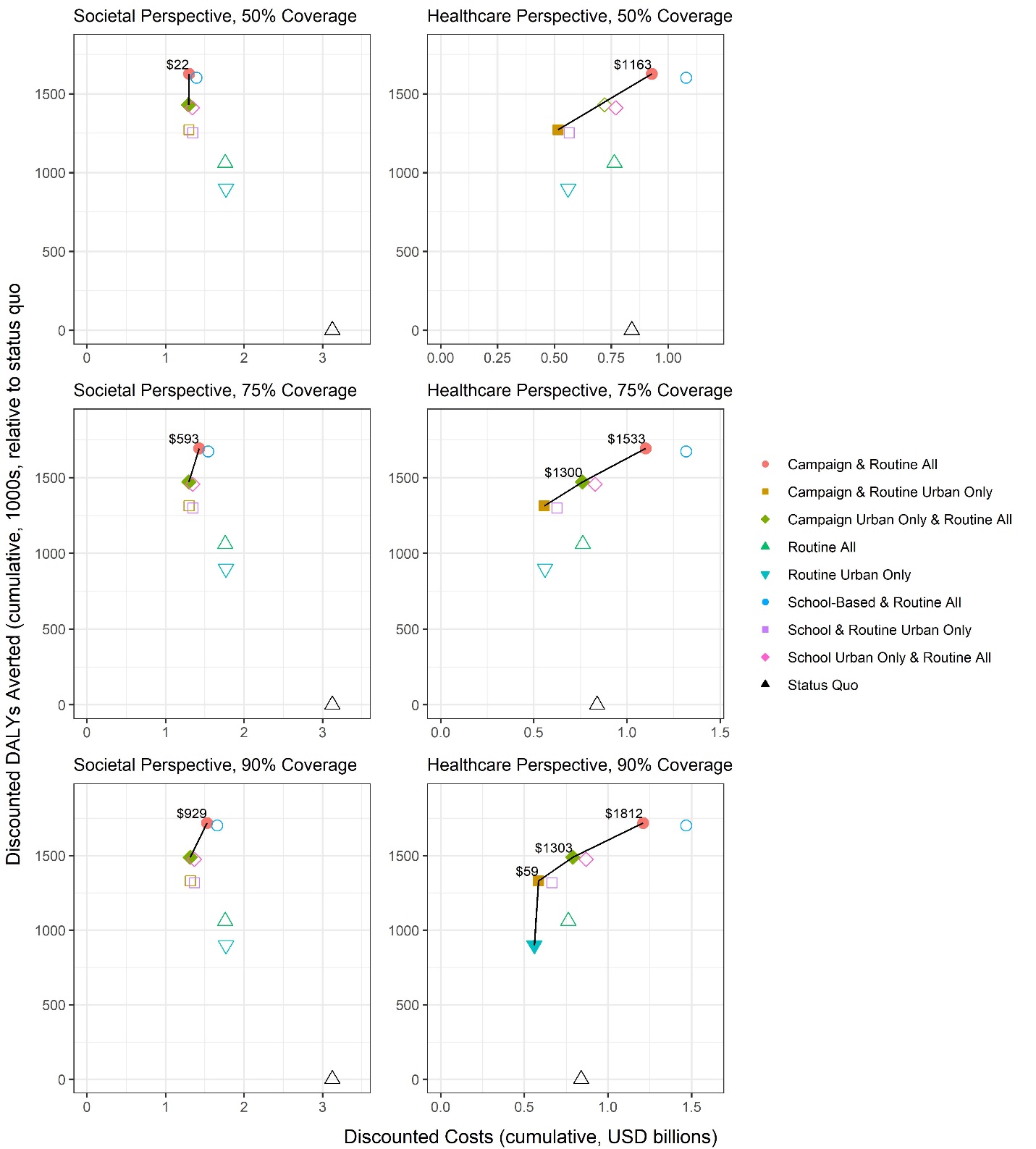
**

Note: Figure shows cumulative discounted costs (USD billions) on the x-axis and cumulative discounted DALYs averted (thousands) compared to the status quo of no national vaccination strategy. Filled markers indicate those on the cost-effectiveness frontier, while unfilled markers indicate that the strategy is dominated (costs more for less health benefit compared to another strategy). Panels vary by vaccine campaign coverage (both school- and community-based; base case is 90%) and perspective (societal or healthcare). All panels show results using a ten-year analytic horizon.

**Supplementary Figure 10: Cost-effectiveness results under alternate vaccine prices**

**
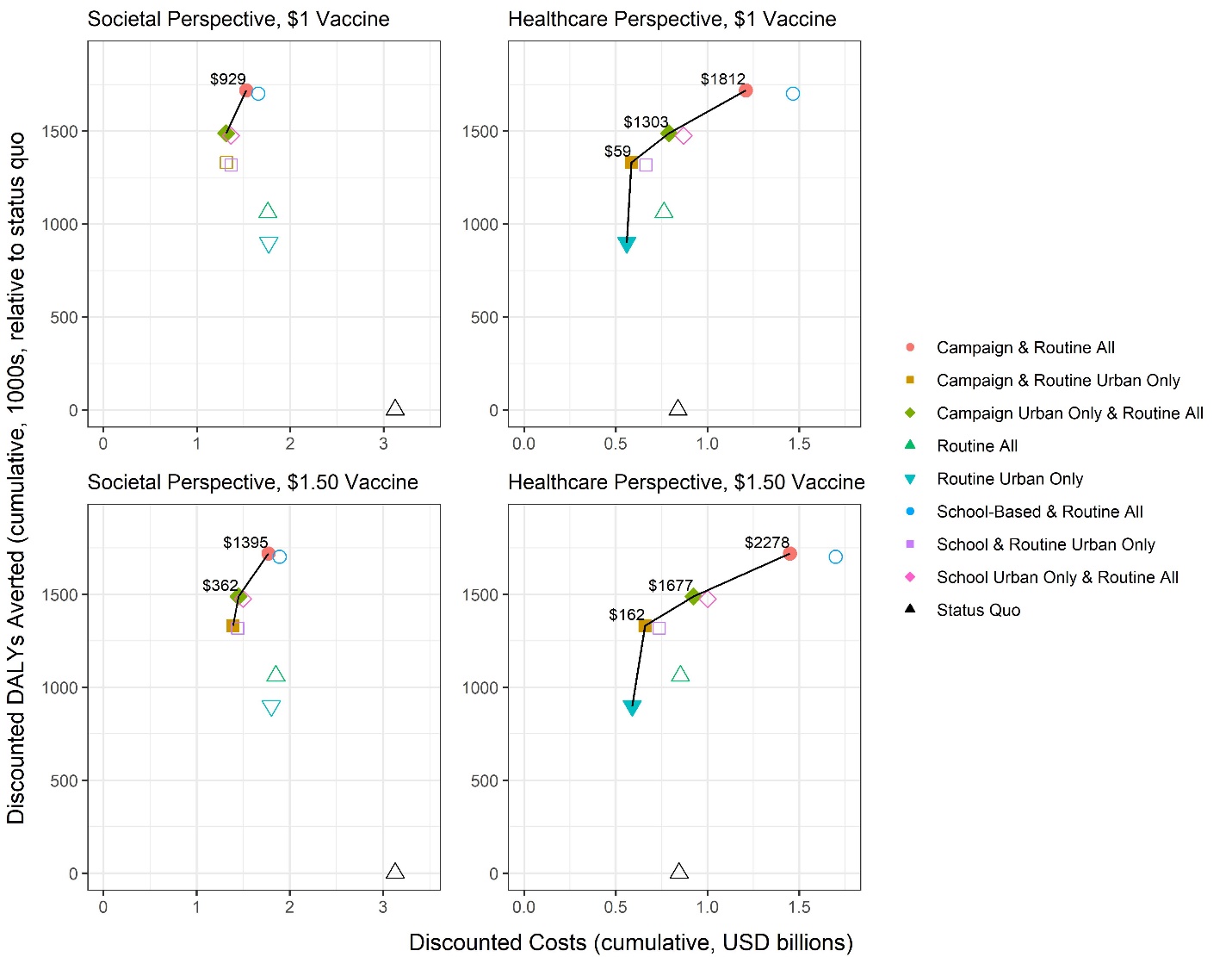
**

Note: Figure shows cumulative discounted costs (USD billions) on the x-axis and cumulative discounted DALYs averted (thousands) compared to the status quo of no national vaccination strategy. Filled markers indicate those on the cost-effectiveness frontier, while unfilled markers indicate that the strategy is dominated (costs more for less health benefit compared to another strategy). Panels vary by the vaccine price (base case/India price is $1, Gavi price is $1.50). All panels show results using a ten-year analytic horizon.

**Supplementary Figure 11: Cost-effectiveness results under more pessimistic vaccine efficacy assumptions**

**
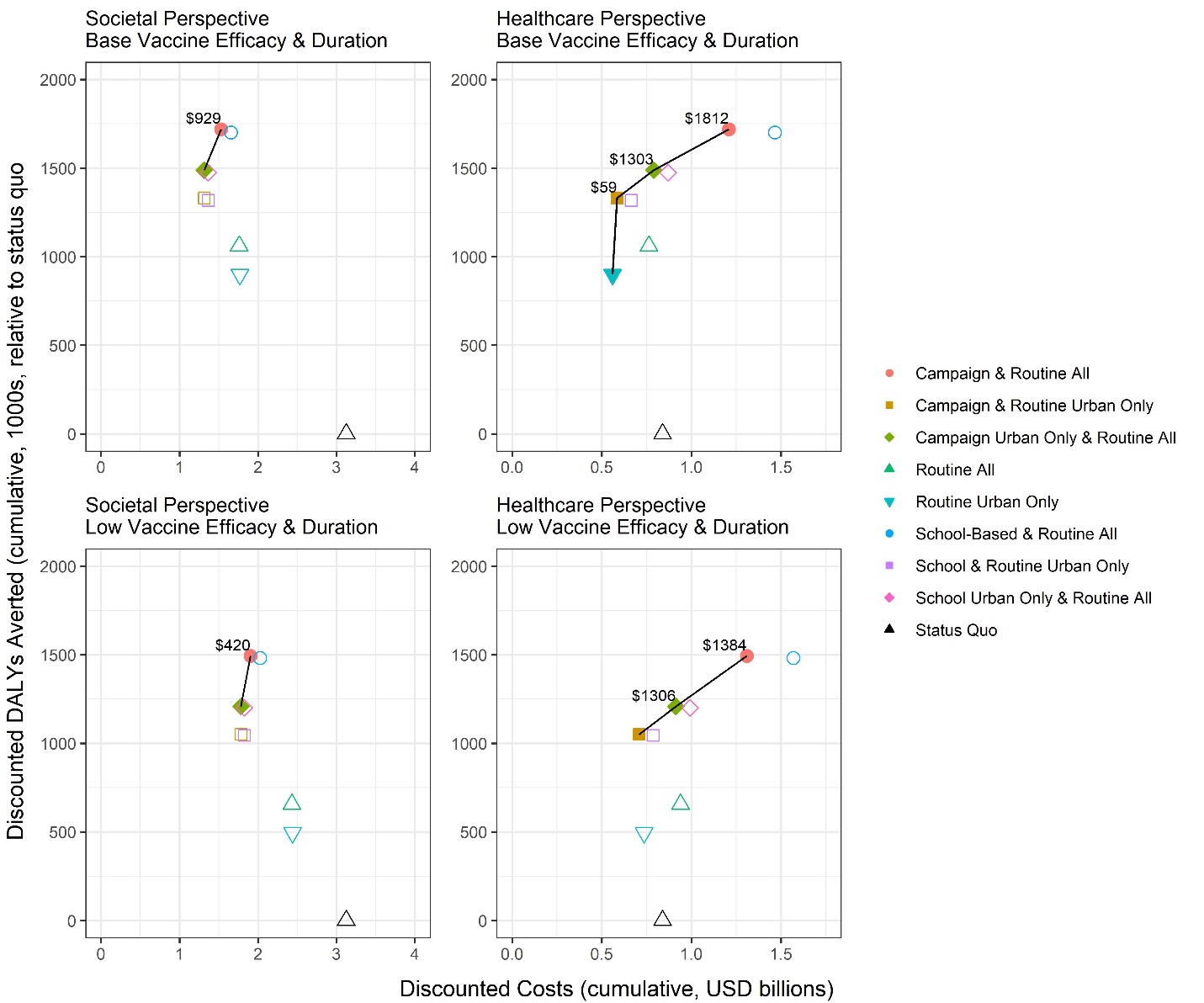
**

Note: Figure shows cumulative discounted costs (USD billions) on the x-axis and cumulative discounted DALYs averted (thousands) compared to the status quo of no national vaccination strategy. Filled markers indicate those on the cost-effectiveness frontier, while unfilled markers indicate that the strategy is dominated (costs more for less health benefit compared to another strategy). The top panel shows outcomes under the base case (vaccine efficacy and duration of immunity from vaccination are both varied in the PSA, with efficacy averaging 82% [59-92%] and duration of immunity averaging 20 years [15-30 years]. The bottom panel shows outcomes under a pessimistic vaccine efficacy scenario, in which vaccine efficacy is fixed at 59% (the lower bound from Shakya et al [24]) and duration of immunity is fixed at 10 years (half the base case average). All panels show results using a ten-year analytic horizon.

**Supplementary Figure 12: Variation in costs and health impact of routine delivery window**

**
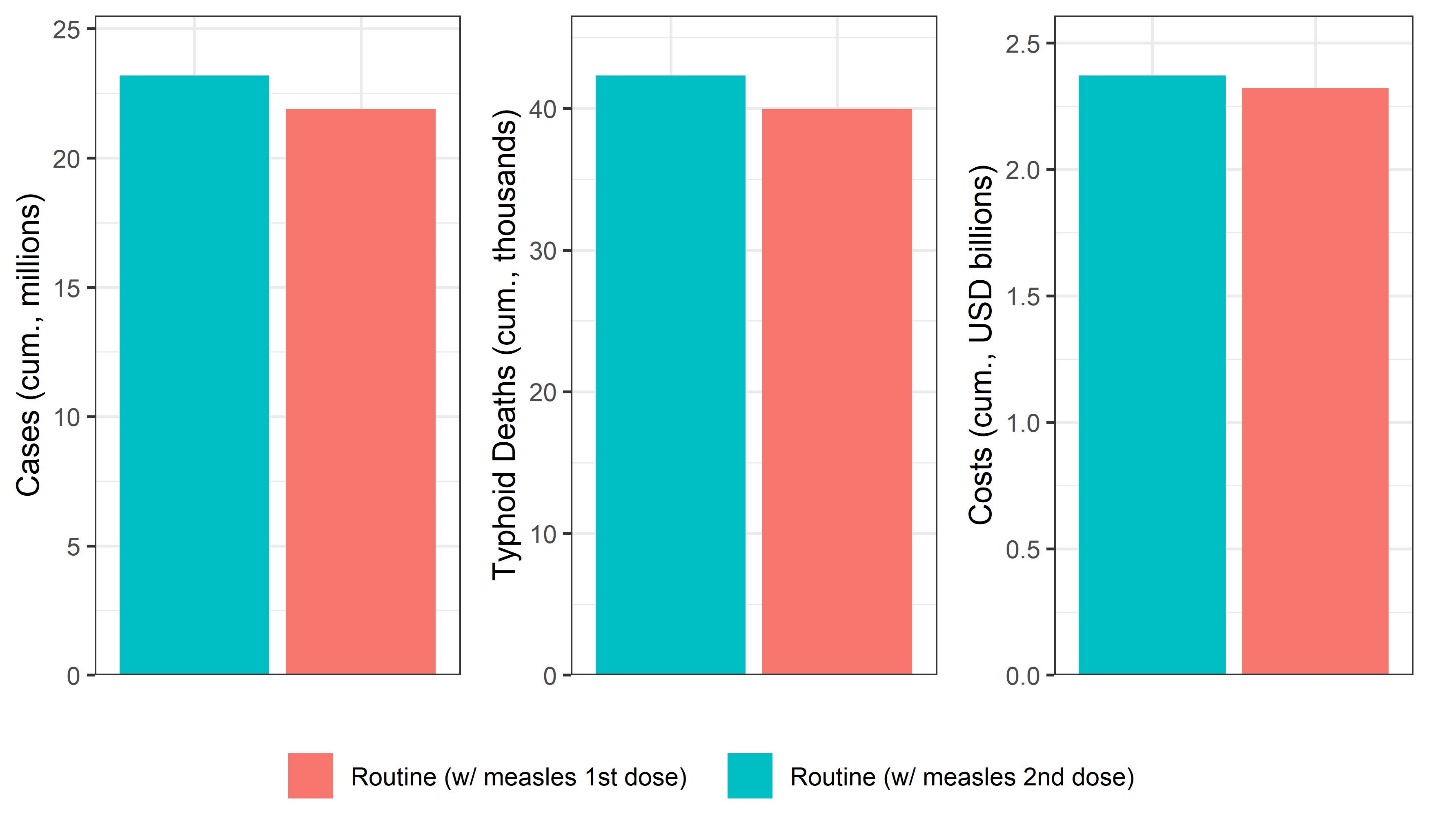
**

Note: Figure compares the 10-year cumulative cases, typhoid deaths, and costs under nationwide routine vaccination, delivered alongside measles first dose (9 months, red) and measles second dose (15 months, blue).

**Supplementary Figure 13: Cost-effectiveness acceptability curve and frontier (healthcare perspective)**

**
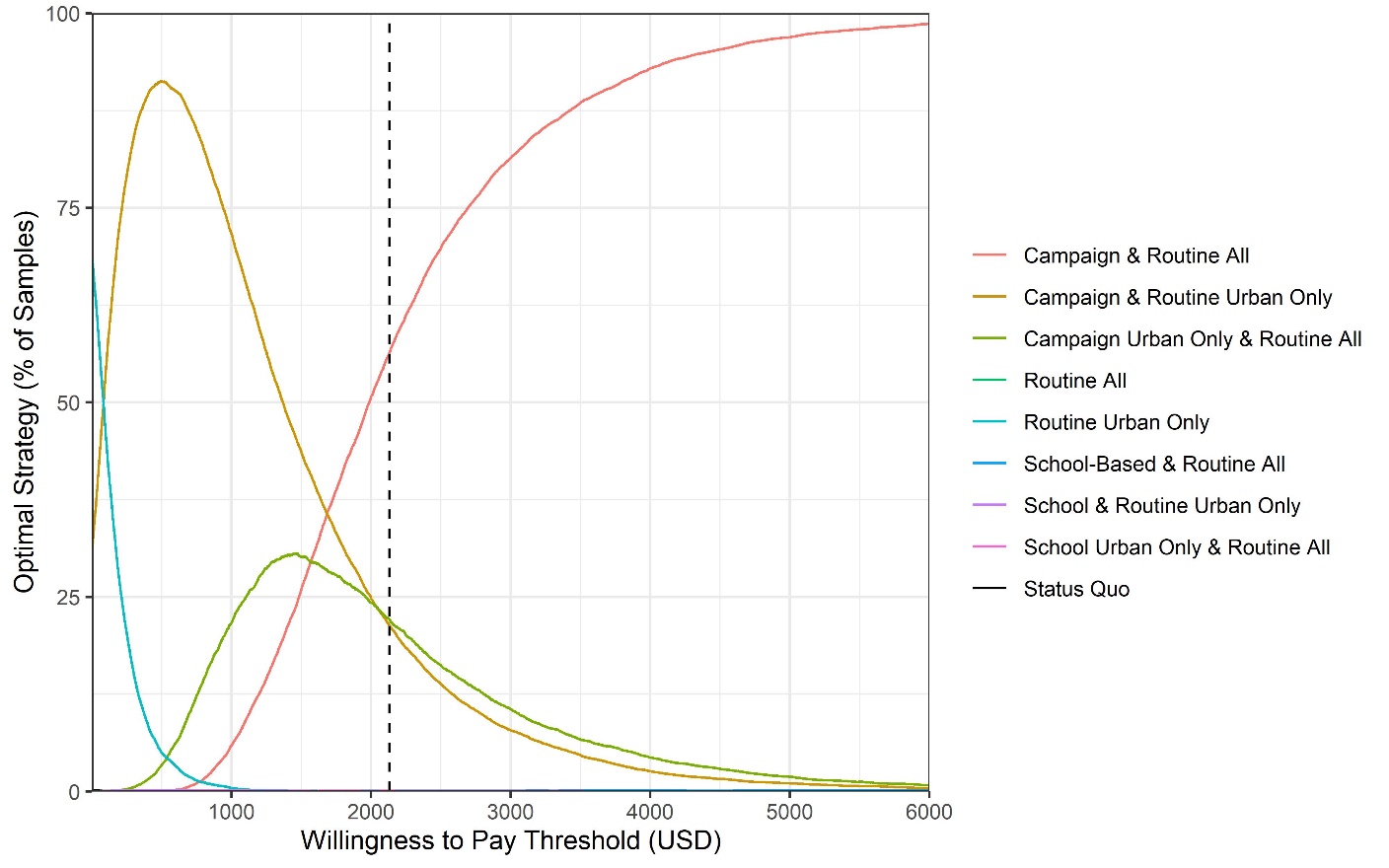
**

**
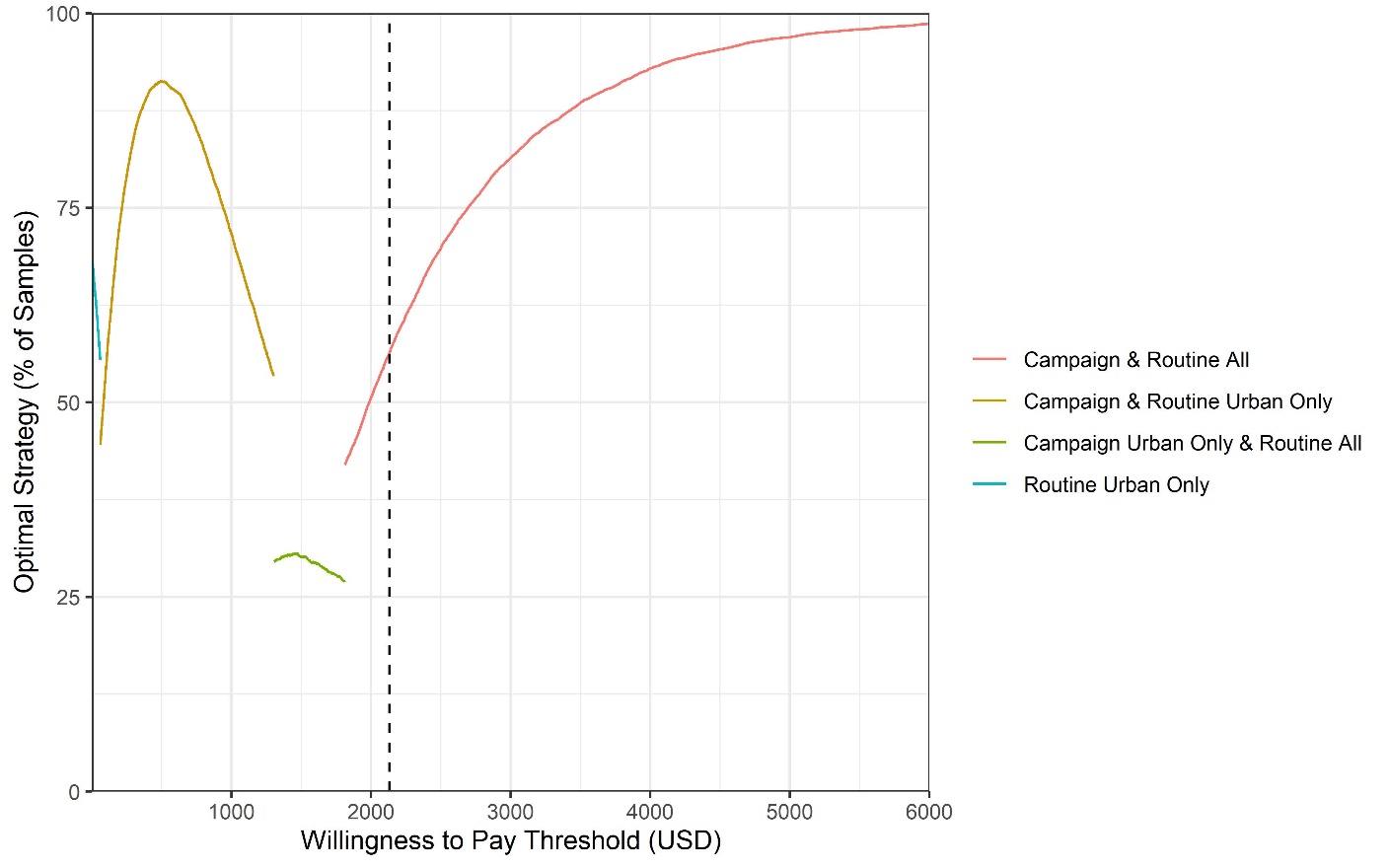
**

Note: Top panel (CEAC) shows the proportion of 10,000 probabilistic sensitivity analysis runs for which a given strategy was considered the preferred strategy (non-dominated with the highest ICER below the willingness to pay threshold) over a range of willingness to pay thresholds. Bottom panel (CEAF) subsets the CEAC to include only those strategies that are considered optimal on expectation at each willingness to pay threshold.

**Supplementary Figure 14: One-way sensitivity analysis (healthcare perspective)**

**
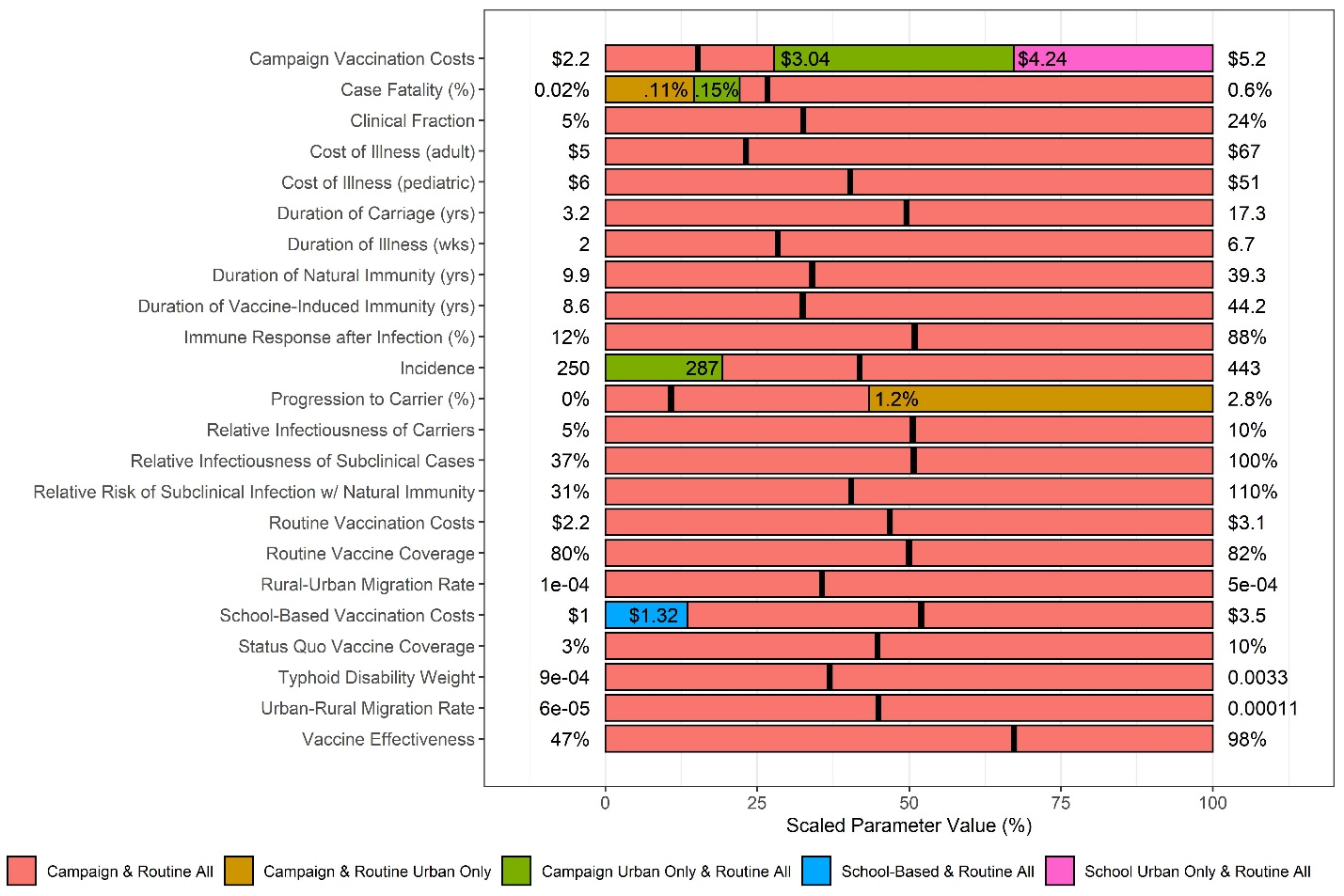
**

Note: Figure depicts the preferred strategy when all parameters are held at their mean values and a single parameter is adjusted over its full range. Thick black lines indicate the mean values for each parameter. The x-axis indicates the parameter value when it is scaled from 0% to 100%, with 0% representing the minimum, 100% representing the maximum, 50% representing the median, and so on. X-axis locations where the graph changes color indicate the threshold of that parameter value at which the optimal strategy changes.

**Supplementary Figure 15: Start-up vaccination costs and affordability**

**
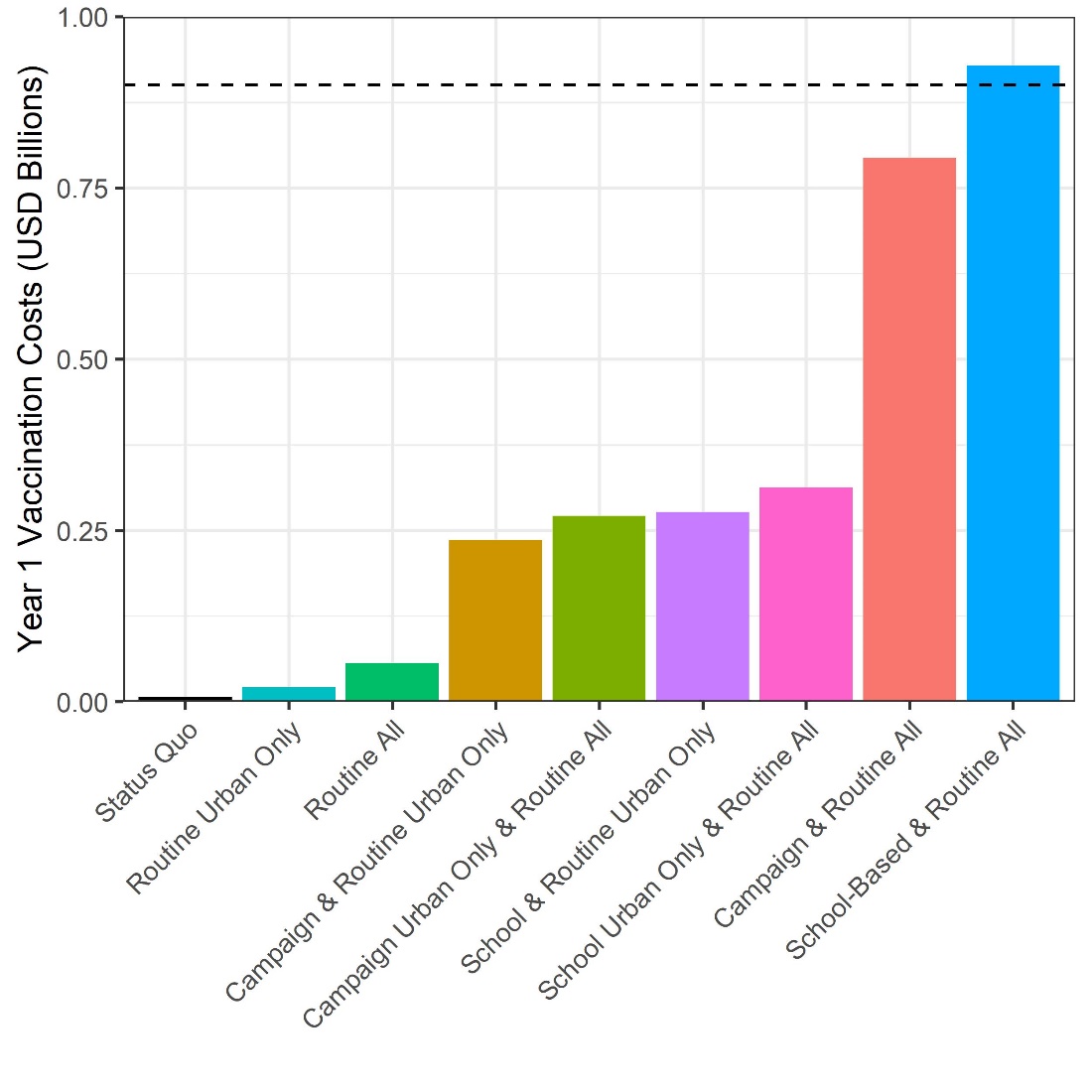
**

Note: Figure shows year 1 vaccination costs (healthcare only) by strategy. The dashed line indicates the Government of India’s vaccine budget from domestic sources in 2017-18 (adjusted to 2019 USD).

1. We omitted Lakshadweep because incidence estimates were not included in [1] [↑](#footnote-ref-1)
